# β-Glucuronidases of opportunistic bacteria are the major contributors to xenobiotic-induced toxicity in the gut

**DOI:** 10.1038/s41598-018-34678-z

**Published:** 2018-11-06

**Authors:** Punsaldulam Dashnyam, Ramesh Mudududdla, Tung-Ju Hsieh, Ting-Chien Lin, Hsien-Ya Lin, Peng-Yuan Chen, Chia-Yi Hsu, Chun-Hung Lin

**Affiliations:** 1 Molecular and Biological Agricultural Sciences, Taiwan International Graduate Program, Academia Sinica and National Chung-Hsing University, Taipei, 11529 Taiwan; 2 Institute of Biological Chemistry, Academia Sinica, Taipei, 11529 Taiwan; 3 Graduate Institute of Biotechnology, National Chung-Hsing University, Taichung, 40227 Taiwan; 4 Biotechnology Center, National Chung-Hsing University, Taichung, 40227 Taiwan; 5 Department of Chemistry and Institute of Biochemical Sciences, National Taiwan University, Taipei, 10617 Taiwan

## Abstract

Gut bacterial β-D-glucuronidases (GUSs) catalyze the removal of glucuronic acid from liver-produced β-D-glucuronides. These reactions can have deleterious consequences when they reverse xenobiotic metabolism. The human gut contains hundreds of GUSs of variable sequences and structures. To understand how any particular bacterial GUS(s) contributes to global GUS activity and affects human health, the individual substrate preference(s) must be known. Herein, we report that representative GUSs vary in their ability to produce various xenobiotics from their respective glucuronides. To attempt to explain the distinct substrate preference, we solved the structure of a bacterial GUS complexed with coumarin-3-β-D-glucuronide. Comparisons of this structure with other GUS structures identified differences in loop 3 (or the α2-helix loop) and loop 5 at the aglycone-binding site, where differences in their conformations, hydrophobicities and flexibilities appear to underlie the distinct substrate preference(s) of the GUSs. Additional sequence, structural and functional analysis indicated that several groups of functionally related gut bacterial GUSs exist. Our results pinpoint opportunistic gut bacterial GUSs as those that cause xenobiotic-induced toxicity. We propose a structure-activity relationship that should allow both the prediction of the functional roles of GUSs and the design of selective inhibitors.

## Introduction

The human gut microbiota, representing a complex and dynamic microbial community in the gastrointestinal tract, co-evolves and co-develops with its host^1^. In this evolutionary niche, the capacity to use various carbon sources provides gut bacteria with competitive advantages that increase their chances for survival and/or abundance^2–4^. For instance, gut microbes often can process host-indigestible plant polysaccharides or host-derived mucin glycans^5–8^. Many gut microbes can also process glucuronides—the end products of glucuronidation—to obtain glucuronic acid. Glucuronidation is a major detoxification pathway in mammalian liver, where UDP-glucuronosyltransferases catalytically conjugate hydrophobic xenobiotics and endobiotics to glucuronic acid, thereby increasing their solubility^9^. Once the resulting glucuronide enters the intestine, a gut bacterial β-D-glucuronidase (GUS) removes the glucuronic acid, thereby releasing the original molecules into the gut lumen^10,11^. Therefore, the activities of these enzymes affect the physiological activities and toxicities of various drugs (e.g. SN38 and NSAIDs) and xenobiotics^12^. Consequently, reactivation of the released xenobiotics and endobiotics results in toxicity (diarrhea or epithelial injury) in the gut and increases the lifetimes of these compounds in the circulation^13–15^. Moreover, the activities of fecal or serum GUSs have been reported to be elevated in patients with colon cancer^16^ and gastrointestinal tract carcinomas^17^. Indeed, gut bacterial GUSs are involved in the toxicity of carcinogenic aromatic compounds. For example, the carcinogenicity of hetero-aromatic compounds that is produced during meat processing was found to be associated with gut bacterial *uid*A (also known as *gus*A)^18^. In addition, bacterial GUS activity in the cecum delays elimination of the endocrine-disrupting agent, bisphenol A^19^.

Despite these detrimental activities, gut bacterial GUSs are essential for recycling of important endogenous molecules and for the regeneration of beneficial natural products^10,20^. The biological activities of lignans, flavonoids (baicalein), ceramides and glycyrrhetinic acid are greater than their corresponding glucuronides^20^. Several types of gut bacteria are able to produce glycyrrhetic acid and glycyrrhetinic acid monoglucuronide from glycyrrhizin, the diglucuronide conjugate of glycyrrhetinic acid^21,22^. The efficacy of ceramide glucuronide against colon cancer has been linked to GUS activity that produces bioactive ceramides in the colon^23^. In addition, GUS activity in children with inflammatory bowel disease was found to be half that found for a control group^24^. These examples illustrate the importance of maintaining a healthy level of gut bacterial GUSs.

These contradicting reports concerning the potential positive and negative effects of GUS activities suggest that the gut bacterial GUSs all display a different spectrum of substrate preferences and that there are specific groups of GUSs for desirable and undesirable activities, with each displaying a unique spectrum of substrate preferences. Gut bacterial GUSs have diverse structures and catalytic efficiencies^25^. A recent work has described the variation in the length of GUS active-site loops that resulted in different active-site architectures and that correlated with differential processing of *p*-nitrophenyl β-D-glucuronide and heparan nonasaccharide in several bacterial GUSs^26^. However, the aglycone moieties of GUS substrates vary greatly in size, polarity, and functionality^10^. Examining how different GUSs process various glucuronides is essential if the functional roles of gut bacterial GUSs are to be understood. Therefore, a variety of xenobiotic glucuronides must be used to examine the activities of different gut bacterial GUSs to better understand the individual roles of the GUSs in GUS-induced pathology. In addition, examination of the structure(s) of a GUS/substrate complex is required to elucidate how different bacterial GUSs process different glucuronides. The ultimate goal is to pinpoint a specific group of GUSs with activities that cause the toxicities of certain drugs or xenobiotics and to then develop inhibitors that selectively target those GUSs.

For the study reported herein, we examined the activities of GUSs from gut bacteria that represent five major groups of gut microbiota, namely *Rg*GUS, *Cp*GUS, *Bf*GUS, *Ec*GUS, and *Bifd*GUS, from *Ruminococcus gnavus* (belonging to Firmicutes), *Clostridium perfringens* (belonging to Firmicutes), *Bacteroides fragilis* (belonging to Bacteroidetes), *Escherichia coli* (belonging to Proteobacteria), and *Bifidobacterium dentium* (belonging to Actinobacteria), respectively. The activities of these GUSs were tested with 22 substrates, including glucuronides derived from natural products, industrial chemicals, and pharmaceutical drugs. Our structural and functional studies indicate that GUSs of opportunistic and enteropathogenic bacteria are the major types of enzymes that regenerate toxic xenobiotics in the gut.

## Results and Discussion

### Five types of gut bacterial GUSs have distinct substrate preferences

**Figure 1:**
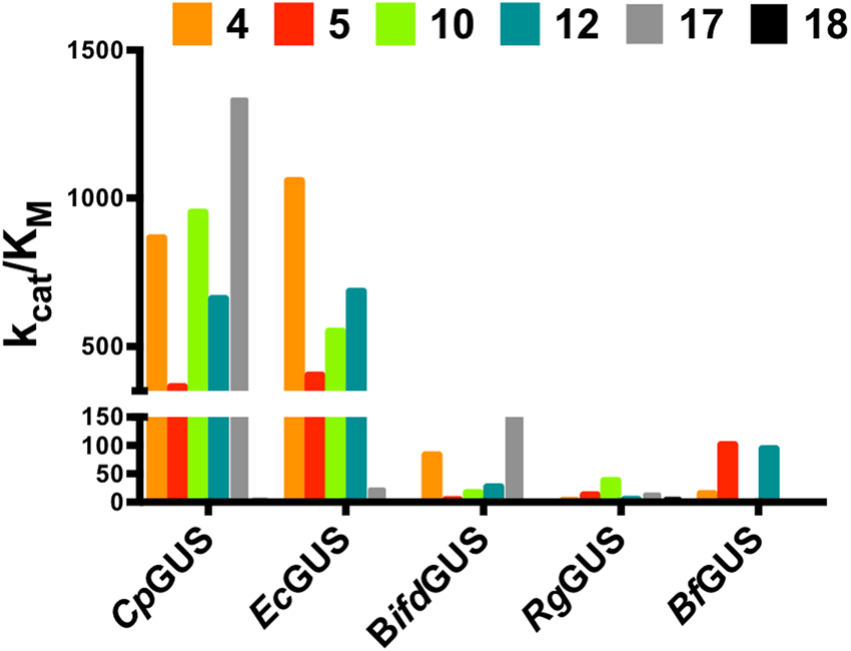
*Cp*GUS and *Ec*GUS exhibit higher catalytic efficiency (k_cat_/K_M_) than do the other GUSs. Comparison of k_cat_/K_M_ values of the five bacterial GUSs used in this study for substrates **4**, **5**, **10**, **12**, **17**, and **18** indicating the superior catalytic efficiency of *Cp*GUS and *Ec*GUS compared with *Rg*GUS, *Bf*GUS, and *Bifd*GUS. See also Supplementary Tables S1 and S2 for the kinetic parameters, and Fig. 2h for the molecular structures of the substrates.

To examine the capacities of gut bacterial GUSs to regenerate xenobiotics from their corresponding glucuronides *in vitro*, we examined the activities of five gut bacterial enzymes (*Ec*GUS, *Cp*GUS, *Rg*GUS, *Bifd*GUS, and *Bf*GUS) for six substrates (**4**, **5**, **10**, **12**, **17**, and **18**). The activity assays were conducted using recombinant enzymes that were expressed in *E. coli* and purified to homogeneity (see Methods). *Cp*GUS and *Ec*GUS exhibited greater catalytic efficiencies (k_cat_/K_M_) toward most of the tested substrates in comparison with *Bifd*GUS, *Rg*GUS, and *Bf*GUS (Fig. 1; see Supplementary Tables S1 and S2). Despite the similar catalytic efficiencies of *Ec*GUS and *Cp*GUS toward smaller substrates, *Ec*GUS displayed much lower activities toward the larger substrates, **17** and **18**. *Bifd*GUS and *Bf*GUS showed a similar trend in kinetics parameters (K_M_ and k_cat_ values), but *Bifd*GUS more efficiently hydrolyzed the bulky substrate **17**. Although *Rg*GUS was a very poor catalyst in general, it hydrolyzed the naturally occurring glucuronide **18** faster than did any of the other enzymes. In general, the K_M_ values for the glucuronides decreased as the size of their aglycones increased. A greater k_cat_ value was found for **4** and **5** probably because the nitrophenylates are a good leaving groups.

**Figure 2:**
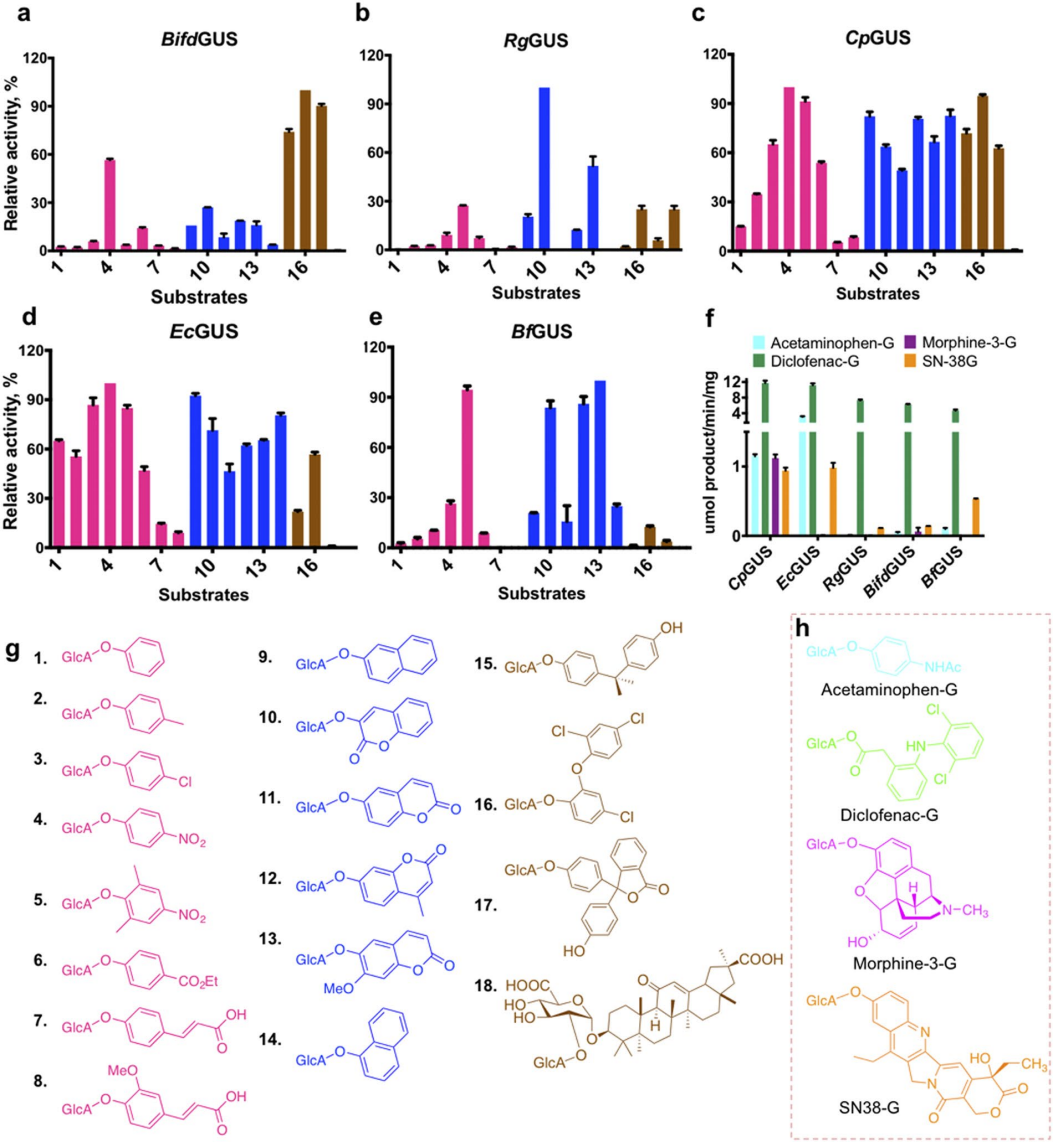
The five tested gut bacterial GUSs have distinct substrate preferences. Substrate preferences of the five gut bacterial GUSs and molecular structures of the glucuronides are shown. Relative activities of (**a**) *Bifd*GUS, (**b**) *Rg*GUS, (**c**) *Cp*GUS, (**d**) *Ec*GUS, and (**e**) *Bf*GUS toward substrates **1**–**18**. The structures of the substrates are shown in (**g**) and were categorized into three groups according to the type of aglycone as mono-aromatic (pink), fused aromatic (blue), and bulky aglycones (brown). Panel **f** compares the specific activities of the GUSs for five drug glucuronides with their structures are shown in panel **h**. Relative activities of the GUSs were calculated as specific activity (μmol/min/mg) and were determined as follows. Enzymes (10 ng of *Cp*GUS and *Ec*GUS, and 50 ng of *Rg*GUS, *Bf*GUS, and *Bifd*GUS) were incubated with 250 μM substrates in 50 mM sodium citrate, 150 mM NaCl at pH 6.0. The highest activities for a given substrate was set as 100%. For *Cp*GUS, *Ec*GUS, *Rg*GUS, *Bf*GUS, and *Bifd*GUS, the greatest activities were for **4**, **10**, **15**, and **16**, respectively. (**g**) Molecular structures of xenobiotic glucuronides used in this study are for: **1**, phenoxy; **2**, *p*-methylphenoxy; **3**, *p*-chlorophenoxy; **4**, *p*-nitrophenoxy; **5**, 2,6-dimethyl-4-nitrophenoxy; **6**, 4-(ethoxycarbonyl)phenoxy; **7**, 4-((*E*)-2-carboxyvinyl)phenoxy; **8**, 4-((*E*)-2-carboxyvinyl)-2-methoxyphenoxy; **9**, naphthalen-2-yloxy; **10**, 2-oxo-2*H*-chromen-3-yloxy; **11**, 2-oxo-2*H*-chromen-6-yloxy; **12**, 4-methylumbelliferyloxy; **13**, 7-methoxy-2-oxo-2*H*-chromen-6-yloxy; **14**, naphthalen-1-yloxy, **15**, bisphenyl-A; **16**, triclosan; **17**, phenolphthalein; and **18**, glycyrrhizin. (**h**) Molecular structures of drug glucuronides.

Humans are often exposed to numerous xenobiotics in their diet, from the environment, and in the form of drugs^27^. Many of these compounds are subjected to β-D-glucuronidation in the liver, yielding a form that can be excreted. To bring increased diversity to substrate chemistry, we synthesized additional 12 β-D-glucuronides according to previously reported methods^28,29^. As shown in Fig. 2g, the 18 substrates could be separated into three groups according to their aglycone size, i.e., as mono-aromatic (pink), fused aromatic (blue), and bulky aglycones (brown). Each GUS included in this study was examined for its capacity to hydrolyze these substrates. Profiling of the GUS-catalyzed reactions indeed resulted in distinct hydrolysis patterns (Fig. 2a–e). Figure 2 displays the relative activities of each GUS to emphasize its substrate preference. *Cp*GUS and *Ec*GUS cleaved many, but not all, of the tested glucuronide substrates with preference for the phenolic and fused phenolic substrates (Fig. 2c,d). However, *Cp*GUS had notably greater activities for **15**–**17** relative to *Ec*GUS. On the other hand, *Rg*GUS showed a substantial preference for the few glucuronides that contained one or more polar functional groups (**10**, **13**, **16**, and **18**) (Fig. 2b). In a similar manner, the preference of *Bf*GUS was limited to only a few substrates, including **5**, **10**, **12**, and **13** (Fig. 2e). *Bifd*GUS obviously favored bulky substrates, although the types of bulky aglycone that could be hydrolyzed by *Bifd*GUS were limited in certain size and shape. For instance, this enzyme could hydrolyze **17**, but not **18** (Fig. 2a).

A high degree of consistency exists between the relative activity and the catalytic efficiency of each GUS, suggesting that the specific activity is a useful measure with which to compare the substrate preference of the GUSs. Overall, these enzymes seem to have differing capacities to process the tested substrates, and their preference is dependent on the size, shape, and polarity of the aglycone of the substrate. Notably, the glucuronides of the industrial chemicals (**6**, **9**, and **14**–**16**) are readily hydrolyzed by all the GUSs, especially by *Cp*GUS and *Ec*GUS, which displayed the greatest ability to hydrolyze the glucuronides of common drugs, such as SN38 and diclofenac (Fig. 2f). In fact, 4-methylumbelliferone (the aglycone of **12**) is an approved drug for biliary spasm in some countries, and its most common side effects are diarrhea and other mild gastrointestinal symptoms^30^. We, therefore, conclude that *Cp*GUS and *Ec*GUS represent an undesirable group of GUSs capable of regenerating toxic xenobiotics in the gut, although we cannot exclude the possibility that enzymes similar to *Bf*GUS, *Rg*GUS, and *Bifd*GUS may also regenerate unwanted xenobiotics in a substrate-dependent manner. This uncertainty warrants the need to develop selective inhibitors for each GUS group.

Based on the observed patterns of substrate preference, selective inhibitors can be designed and synthesized as long as UIFG (which potently, yet broadly, inhibits bacterial and mammalian GUSs^31^) is attached to a suitable aglycone moiety. In our preliminary screening, UIFG universally inhibited all tested GUSs, but the incorporation of a propyl group at the C6 position (UIFG-Pr) selectively targeted *Cp*GUS and *Ec*GUS because 1 μM of UIFG-Pr suppressed about ~80–90% of *Cp*GUS and *Ec*GUS activities; conversely the other enzymes were only slightly inhibited at the same concentration of UIFG-Pr (see Supplementary Fig. S1). These results indicated that the addition of different substituents one at a time onto IUFG is a promising approach for development of selective and potent inhibitors for different GUSs.

### Loops 3 (or α2-helix loop) and 5 dictate substrate preference of GUSs

To explain the observed patterns of substrate preferences, we compared the crystal structures of the five enzymes. We solved the complex structures of *Bifd*GUS^E479A^/**10**, *Rg*GUS/UIFG, and *Bf*GUS/UIFG (see Methods). The structures of *Cp*GUS (PDB code: 4JKM) and *Ec*GUS (PDB code: 3LPG) are available in PDB. These GUSs exist as homotetramers, and share a similar overall tertiary structure comprised of an N-terminal jelly-roll domain, a middle region that has a immunoglobulin heavy chain-like structure, and a C-terminal triosephosphate isomerase (TIM) barrel domain (see Supplementary Fig. S2); notably *Bf*GUS contains an additional carbohydrate-binding domain at its C-terminus. As with all glycoside hydrolase family 2 enzymes, the active sites of the GUSs are located at the center of the TIM barrel, which consists of eight β-sheets and eight α-helices connected by α/β and β/α loops. Certain β/α loops form the active site, and for purposes of this discussion we numbered them from 1 to 8 starting from the N-terminus (see Supplementary Fig. S2).

The overall structures of currently available glycoside hydrolase family 2 GUSs are similar. Root-mean-square deviations (RMSDs) of the pairwise comparisons of the 193 to 259 equivalent Cα atoms in the TIM barrel domain of the five GUSs are between 0.468–2.326 Å. However, notable structural differences exist near their active sites. A hairpin insertion in the jelly-roll domain is conserved in *Rg*GUS, *Cp*GUS, and *Ec*GUS, whereas it is replaced by a helix structure (namely α2-helix) in *Bifd*GUS. Intriguingly neither the hairpin nor the helix is conserved in *Bf*GUS (see Supplementary Fig. S6). The hairpin insertion is important for targeting of the human GUS to the lysosome^32,33^, but why only some bacterial GUSs contain this motif is not clear.

**Figure 3:**
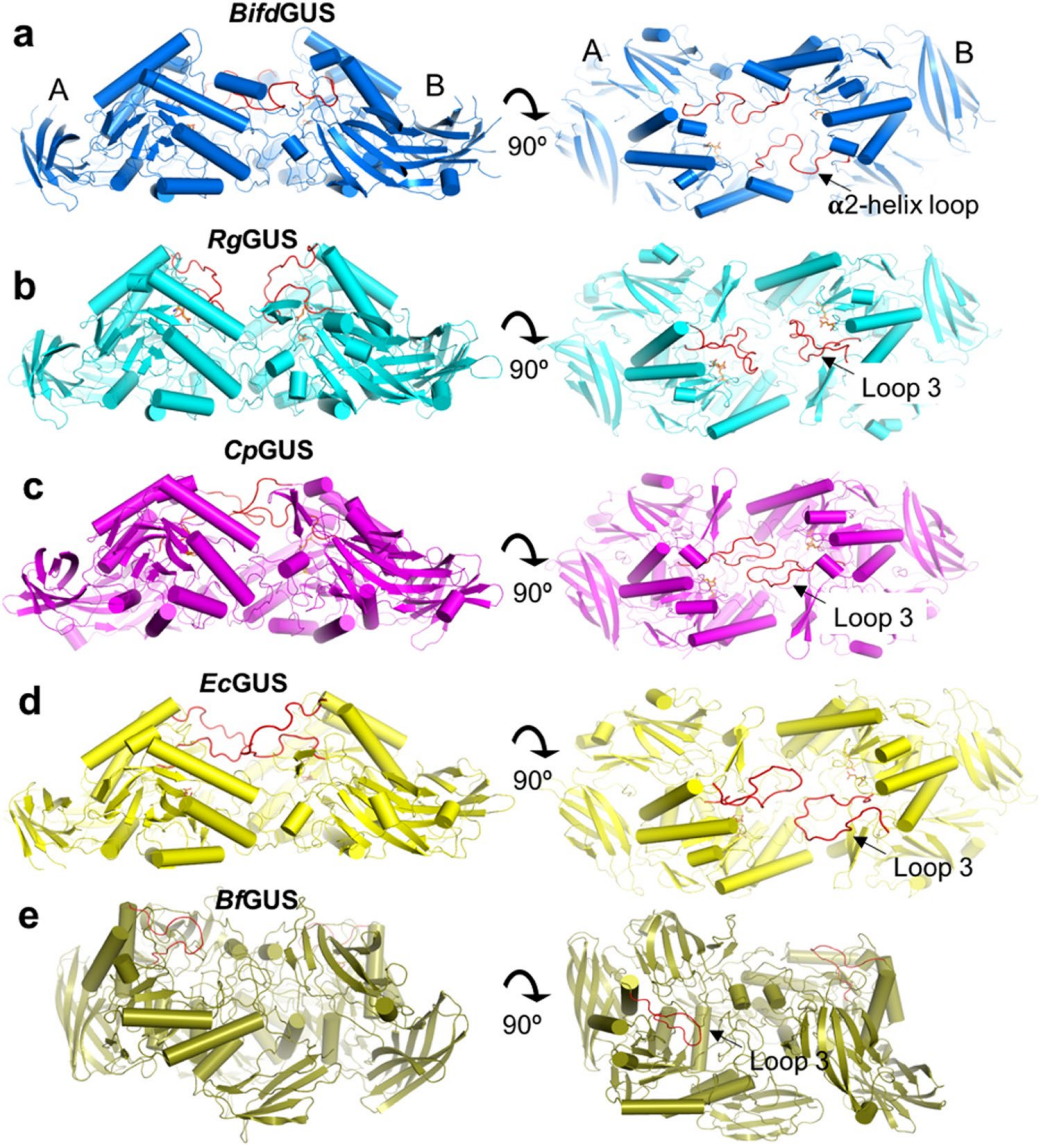
Comparison of the dimeric structures of the five gut bacterial GUSs. Top and side views of the dimers of the tetrameric (**a**) *Bifd*GUS, (**b**) *Rg*GUS, (**c**) *Cp*GUS (**d**) *Ec*GUS and (**e**) *Bf*GUS. The highlighted loops (red) are located either at the periphery of the TIM barrel (loop 3) or at the jelly-roll domain (α2-helix loop). (**a**) Each α2-helix loop in *Bifd*GUS stretches to the ABS of the adjacent subunit. Each loop 3 of *Cp*GUS (**c**) and *Ec*GUS (**d**) exists in an open form and extends from its own subunit to the ABS of the adjacent subunit. Conversely, both loops 3 are not in contact with the adjacent subunit in *Rg*GUS (**b**). The dimeric interface of *Bf*GUS (**e**) is oriented differently than in the other GUSs as the active site of the two monomers do not face each other. See also Supplementary Fig. S2. Structural representations were prepared with the use of the Pymol Molecular Graphics System (version 1.8.4.0 Schrodinger).

A second important structural difference is that loop 3 of the TIM barrel is longer in *Cp*GUS, *Ec*GUS, and *Rg*GUS (26–27 residues), but is shorter in *Bf*GUS (15 residues) and *Bifd*GUS (11 residues). The variation in length of loops 3 and 4 (annotated as loops 1 and 2 previously by Pollet and co-workers^26^) shapes the active-site architecture of bacterial GUSs^26^. A long loop 4 is not found in any of the GUSs discussed herein. Loop 3 locates at the dimeric interface of the GUSs, and is important for the activity of *Ec*GUS^10^. Therefore, we compared the dimeric structures of the tetrameric GUSs to examine how loop 3 shapes the aglycone-binding sites (ABSs) of GUSs. Figure 3 shows the orientations and conformations of loop 3 in the five GUSs. The loop adopts a similar orientation in *Cp*GUS and *Ec*GUS, extending to the periphery of the ABS in the adjacent subunit (Fig. 3c,d). Their ABS structures are similar, which is consistent with the aforementioned similarities in the substrate preferences of these enzymes (Fig. 2c,d). Notably, loop 3 exhibits a closed conformation in the *Rg*GUS-UIFG structure, whereas it is disordered in the apo *Rg*GUS structure (Fig. 3b, see Supplementary Fig. S4), indicating that the loop is stabilized upon ligand binding. Conversely, the architecture of the ABS in *Bf*GUS is dissimilar because its loop 3 is short. Unlike the other GUSs, the TIM barrel domains of the two neighboring subunits in *Bf*GUS do not face each other (Fig. 3e). Interestingly, in *Bifd*GUS, the loop that originated from α2-helix (denoted the α2-helix loop) is situated as loop 3 in the other GUSs and also extends into the ABS of the adjacent subunit (Fig. 3a). Taken together, the structural information gleaned from our examination of the crystal structures revealed that loop 3 or the α2-helix loop is critical to the shape of the overall architectures of the ABSs in bacterial GUSs.

To precisely specify the structure of the ABS, we analyzed the structure of a catalytically inactive mutant of *Bifd*GUS in complex with coumarin-3-glucuronide (*Bifd*GUS^E479A^/**10**). The structure of the complex shows that the enzyme uses several highly conserved residues, e.g., D179, H413, N489, W619, N636, and K638, to form hydrogen bonds and electrostatic interactions with the glucuronic acid moiety of the substrate. The aglycone moiety of the substrate makes non-polar contacts with loops 5 and 6, and the two α2-helix loops extended from N-terminal domains of the two adjacent subunits (shown as A and B subunits in Supplementary Fig. S3). Specifically, the side chains of Y539 and Y543 of loop 6 and C518 and Q519 of loop 5 interacts with the aglycone by π-π stacking and non-polar interactions, respectively. The aglycone in subunit A makes hydrophobic contacts with F152 and M153 in the α2-helix loop of subunit A and with N157 of subunit B. Additionally, the main-chain nitrogen of Q519 forms a hydrogen bond with the carbonyl oxygen of the aglycone (see Supplementary Fig. S3).

**Figure 4:**
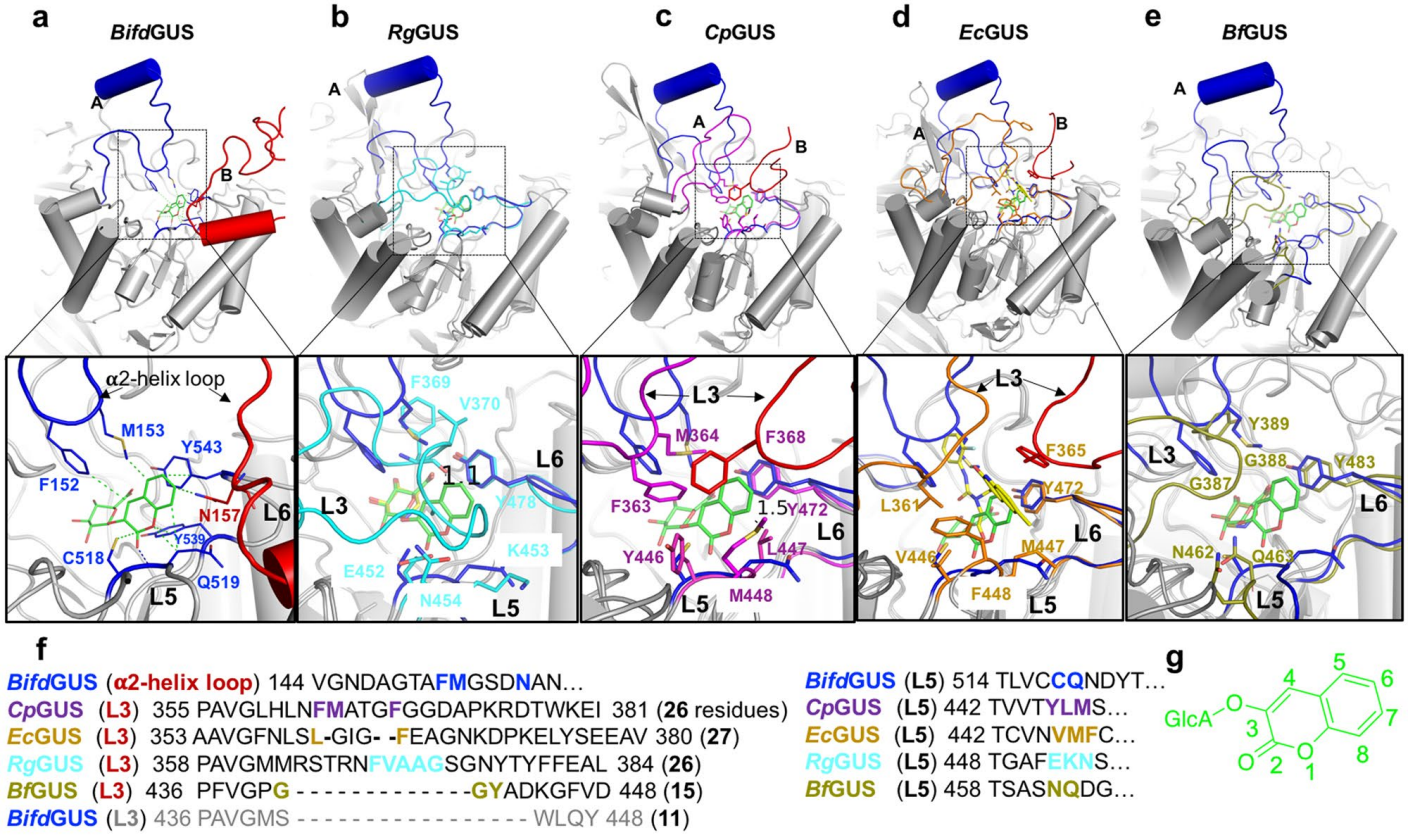
Loop 3 (or the α2-helix loop) and loop 5 are important for the substrate preferences of the GUSs. Comparison of the ABSs of the five bacterial GUSs. The structures of *Rg*GUS/UIFG (**b**), 4JKM (*Cp*GUS-apo) (**c**), 3LPG (the *Ec*GUS/N-substituted-thiourea derivative) (**d**), and *Bf*GUS/UIFG (**e**) were superimposed on the structure of *Bifd*GUS^E479A^/**10** (**a**). The substrate-binding sites of GUSs are located at the center of the TIM barrel domains. Panels (**a**–**e)** display the ABSs that are formed by the conserved loop 6 (L6) and variable loop 5 (L5) and loop 3 (L3) or α2-helix loop. The α2-helix loop in *Bifd*GUS occupies a position similar to that of loop 3 in the other GUSs. (**f**) Sequences of loop 3 (or the α2-helix loop) and loop 5 in the five bacterial GUSs. Residues interacting with the aglycone of **10** are shown as blue, cyan, magenta, yellow, and olive sticks in panels **a**–**e**, respectively. (**g**) Molecular structure of **10**.

We next used the *Bifd*GUS^E479A^/**10** structure as a reference to analyze the ABS structures of the other four GUSs. Noticeable structural differences exist among the ABSs of the *Rg*GUS/UIFG, *Cp*GUS, *Ec*GUS, and *Bf*GUS/UIFG when they were each superimposed on *Bifd*GUS^E479A^/**10**. The ABSs of the aforementioned four enzymes are composed of loops 3, 5 and 6 (Fig. 4b–e). As mentioned earlier, the α2-helix loop is only present in *Bifd*GUS, but not in the other GUSs. The sequence of loop 6 is highly conserved among the GUSs, but those of loops 3 and 5 are not (see Supplementary Fig. S6); therefore, we mainly focused on loops 3 and 5 to investigate if their sequence variations are linked to their distinctive substrate differences. The loops at the ABS region in *Rg*GUS contain both aromatic and small aliphatic residues (F369, V370, A371, A372 in loop 3), and charged residues (E452 and K453 in loop 5; Fig. 4b,f). In *Bf*GUS, these residues are G387, G388, and Y389 (loop 3), and N462 and Q463 (loop 5; Fig. 4e,f). Conversely, the residues of loops 3 and 5 in *Ec*GUS and *Cp*GUS are more hydrophobic and bulkier, i.e., F363, M364, Y446, and L447 in *Cp*GUS and L361, V446, and M447 in *Ec*GUS. Additionally, F368 of *Cp*GUS and F365 of *Ec*GUS are located at the tip of loop 3 and protrude into the binding-site of the adjacent subunit (Fig. 4c–f). This arrangement decreases the volume of the substrate-binding site. Conversely, *Bifd*GUS and *Bf*GUS have a more open and larger ABS space (Fig. 4a,e and Supplementary Fig. S4). Taken together, loop 3 (or the α2-helix loop in *Bifd*GUS) and loop 5 account for the structural variations in the ABS of the GUSs, and are, therefore, responsible for their substrate preferences.

Differences in hydrophobicity are apparent for loop 5 in the GUSs. Loop 5 of *Rg*GUS contains polar and charged residues, and the enzyme readily hydrolyzed **18** (K_M_ 117.6 ± 4.4 uM; k_cat_ 0.66 ± 0.04 sec^−1^). *Rg*GUS efficiently hydrolyzed **18** (glycyrrhizin) to produce glycyrrhetic acid monoglucuronide by removing one glucuronic acid, but not a second glucuronic acid, to produce 18β-glycyrrhetinic acid. After 24 h incubation of **18** with *Rg*GUS, 85% of the glycyrrhizin had been converted into glycyrrhetic acid monoglucuronide. In contrast, loop 5 in *Cp*GUS contains hydrophobic residues, and the enzyme was hardly active for **18**. We replaced the three loop 5 residues YLM (446–448) in the ABS of *Cp*GUS with the corresponding three residues EKN (452–454) in *Rg*GUS. The resulting mutant, *Cp*GUS^*Rg*GUSL5^, had decreased activity for **4**, **12**, and **17** as compared with wild-type *Cp*GUS, although it more efficiently hydrolyzed **18** (see Supplementary Table S1). Given these results, we consider these loop-5 residues to be essential for substrate binding. Consistent with this conclusion, the *Aspergillus oryzae* GUS has been shown to more efficiently catalyze the formation of glycyrrhetic acid monoglucuronide from **18** when the loop 3 and loop 5 residues, corresponding to E452 and V370 in *Rg*GUS, respectively, were replaced with polar residues, such as histidine and glutamine^34^.

Although *Cp*GUS and *Ec*GUS had similar preferred substrates, *Cp*GUS was the superior catalyst in terms of its ability to hydrolyze the bulky substrate **17**, which may be a consequence of the presence of F448 or M448 in loop 5 of *Ec*GUS and *Cp*GUS, respectively. Indeed, the *Cp*GUS^M448F^ mutant had a significantly smaller k_cat_ value for substrate **17** but not for **4** and **12** (see Supplementary Table S1).

### Loop 3 provides the necessary flexibility in the ABS of the bacterial GUSs to accommodate various substrates

**Figure 5:**
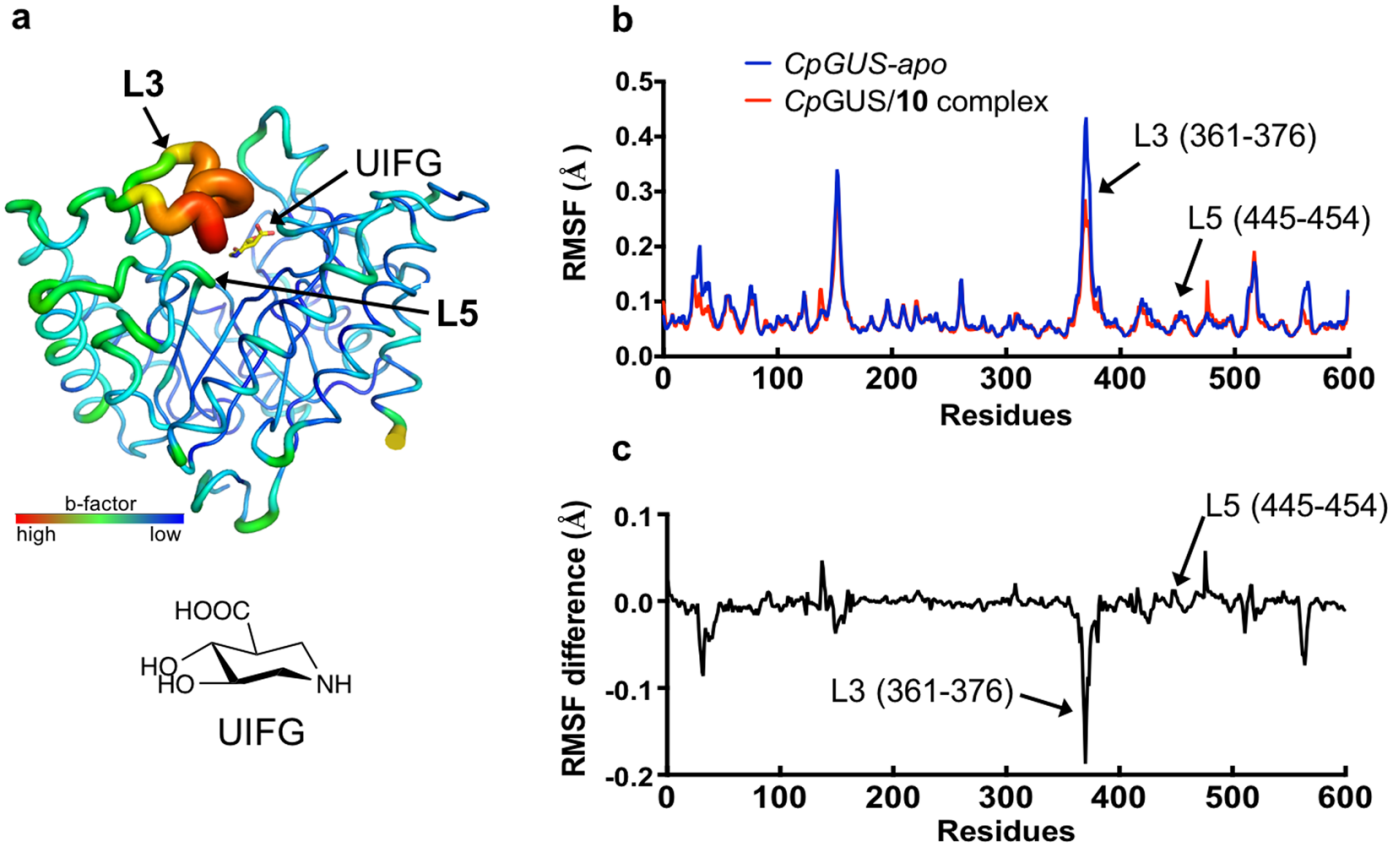
Loop 3 provides necessary flexibility in the ABS of bacterial GUSs. (**a**) The B-factor putty image of the TIM barrel domain of the *Rg*GUS/UIFG complex and the molecular structure of UIFG. The relatively large B-factor of loop 3 in *Rg*GUS indicates a large degree of flexibility. (**b**) The RMSFs of the Cα atoms of apo *Cp*GUS and the *Cp*GUS/**10** complex are shown for 20-ns MD simulations. (**c**) The differences in the RMSFs were calculated the Cα atoms in apo *Cp*GUS and the *Cp*GUS/**10** complex. Loop 3 is the most mobile loop in apo *Cp*GUS and the *Cp*GUS/**10** complex. The RMSF difference of the two forms of *Cp*GUS suggests that the flexibility of loop 3 decreased upon substrate binding. Although the Cα atoms of *Cp*GUS loop 5 seem mobile, side-chain movements of loop 5 residues were observed during the MD simulations.

According to the enzyme-activity analysis, all GUSs accepted one or more substrates larger than **10**, but the superimpositions of *Cp*GUS and *Rg*GUS with the *Bifd*GUS^E479A^/**10** complex indicated possible steric clashes between the aglycone and loop 5 or loop 3 of *Cp*GUS and *Rg*GUS, respectively. Specifically, the backbone carbonyl oxygen atom of F368 of *Rg*GUS is 1.2 Å away from the C5 of the aglycone (Fig. 4b). In addition, side chain of L447 in loop 5 of *Cp*GUS and the C8 of the aglycone are separated by only 1.5 Å (Fig. 4c). Possibly, steric clashes are eliminated by conformational rearrangements in the loops. Indeed, loop 3 is disordered in the apo *Rg*GUS structure (see Supplementary Fig. S4). In the structure of the *Rg*GUS/UIFG complex, the loop was found in a closed conformation and its crystallographic B-factor value is higher than those of other parts of the protein (Fig. 5a). These observations indicate that loop 3 is highly flexible and likely changes its conformation in response to the size and shape of the aglycone. Opening and closing the loop may be required during substrate entry and product exit. Conversely, the crystallographic B-factor of loop 5 is much smaller than that of loop 3 (Fig. 5a), suggesting that any conformational change in loop 5 would be restricted to side chain movement(s).

We expected loop 3 of *Cp*GUS, *Ec*GUS, and *Rg*GUS to adopt different conformations (open and closed or semi-closed) in the complex structures formed with different ligands. However, it is experimentally challenging to prove the existence of such conformations. Therefore, to probe the flexibility of this loop, we performed molecular dynamics (MD) simulations on the apo *Cp*GUS and the *Cp*GUS/**10** complex. The *Cp*GUS/**10** complex was obtained by docking **10** to the 4JKM structure using Autodock vina 1.1.2^35^. The *Cp*GUS dimer was used for the simulations to observe how each loop 3 of the adjacent subunits interact with the aglycone. MD simulations over 10 ns indicated that loop 3 is the most flexible part of the protein as backbone atoms of the loop had the greatest root mean square fluctuations (Fig. 5b). The flexibility of this loop decreased when the substrate was bound to the enzyme (Fig. 5c). Moreover, the side chains of loops 3 and 5 residues were mobile. For example, the distance between C6 and C7 of aglycone to F363 (loop 3) of the A chain and F368 of B chain fluctuated from ~3 to ~13 Å during the simulations (see Supplementary Fig. S5). These results indicated that the ABS of *Cp*GUS is highly flexible, allowing for accommodation of various substrates. It is commonly believed that enzyme active-site loops provide the necessary plasticity so that the active site can adopt different conformations during the substrate entrance, catalysis, and product release. Various studies have demonstrated that increased flexibility of a loop or a side chain improves catalytic efficiency^36–41^. We, therefore hypothesize that enzymes such as *Cp*GUS and *Ec*GUS may have a larger range of conformational dynamics than do the other GUSs to ensure higher catalytic efficiency and a broad substrate preference.

### Structure-guided sequence analysis of the ABSs suggests a functional classification for gut bacterial GUSs

**Figure 6:**
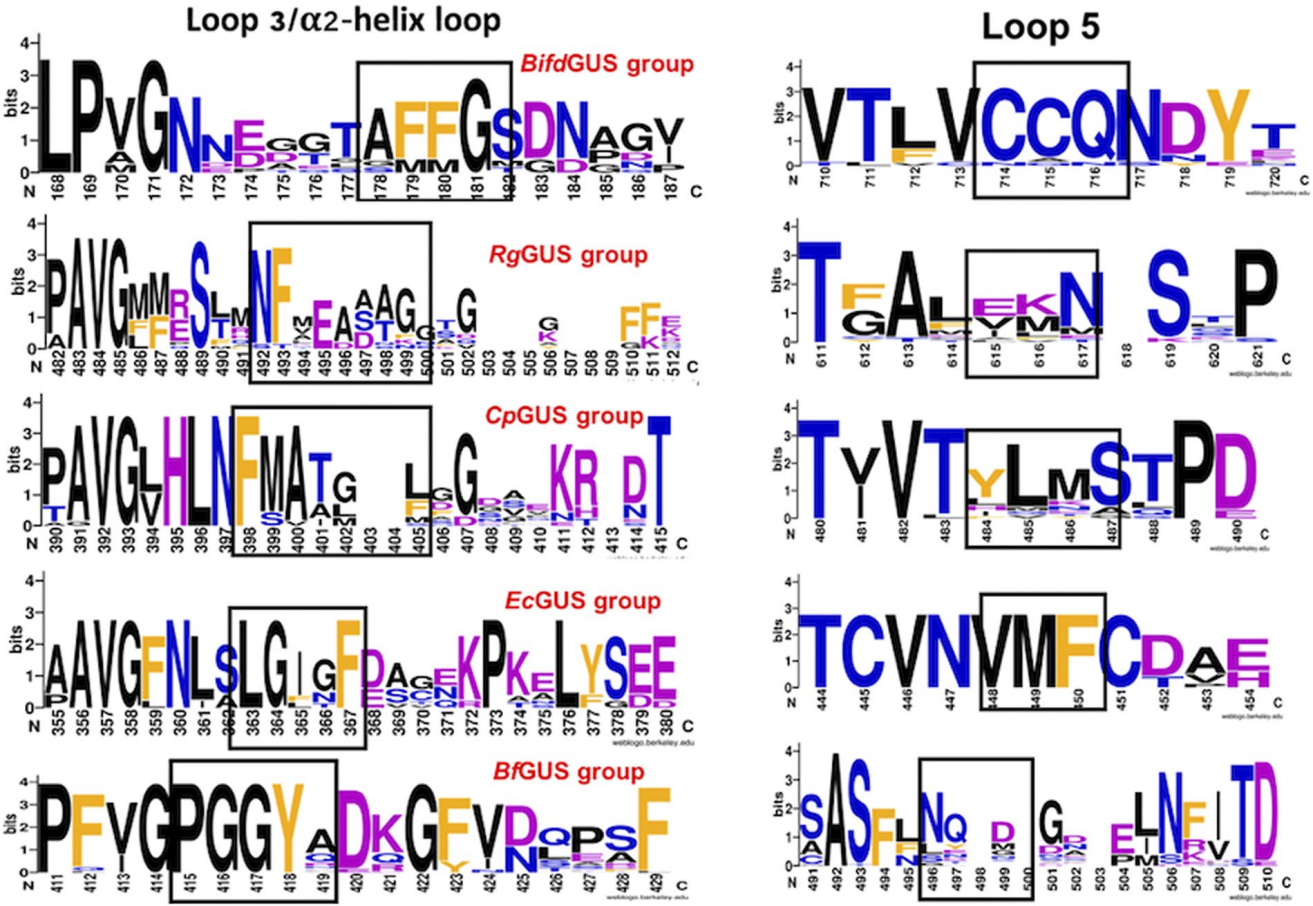
Structure-guided sequence analysis of the ABSs suggests a functional classification for gut bacterial GUSs. Relative occurrence of residues in loop 3 (or in the α2-helix loop) and loop 5 of the bacterial GUSs. Each of the five enzymes studied for this report was used as a query for an NCBI BLASTp search. After downloading the sequences sharing 55 to 95% identity, proteins derived from environmental or industrial sample were manually removed. The remaining enzymes were aligned in the T-Coffee-Expresso server, and a sequence logo for each group was created using WebLogo. The residues forming the ABS of each groups of GUSs are highlighted in black rectangles. The physicochemical properties of the side chains of the residues are shown in black (nonpolar, aliphatic residues), orange (aromatic), blue (polar, uncharged), and purple (polar, charged).

Our studies indicated that different gut bacterial GUSs have evolved so that their ABSs are specific for sets of structurally similar substrates. To further examine the functional and structural diversity of GUS ABSs, we surveyed the relative frequency residues found in the ABS sequences in gut bacterial GUSs sequences. We used BLASTp and each of the five GUS sequences to search for similar GUS sequences in the non-redundant NCBI protein sequence database. All GUS sequences having between 55 and 95% identity were downloaded except for those GUS sequences of environmental- or industrial-related bacteria. The resulting sequence groups were aligned and a sequence logo for each enzyme category created (Fig. 6). Based on the structural information, the residues that may be present as part of the ABS structure and functionality are marked with black rectangles, for the following discussion, only these residues are included. For each group of enzymes, the ABS sequences are similar. Although *Bifidobacterium* belongs to phylum Actinobacterium, the sequences of loops 5 and α2-helix in *Bifd*GUS were well conserved with those of the major Firmicutes bacteria found in the gut, e.g., those of *Roseburia intestinalis*, *R. hominis*, and *Faecalibacterium prausnitzii* (see Supplementary Fig. S7). In contrast, the *Bf*GUS group contained only Bacteroidetes members, and the sequence variation of loops 3 and 5 was relatively small for this group. The sequences of loops 3 and 5 in the *Ec*GUS group were well conserved in the GUSs of a few closely related enteropathogenic bacteria including *Shigella* and *Salmonella* (see Supplementary Fig. S7). Conversely, the loop 3 sequences of the *Cp*GUS and *Rg*GUS groups were more variable and had different number of residues in the loop, which caused several gaps in the respective sequence logos. Although the two groups contained a conserved **NF** motif in loop 3, there were six residues between **AVG** (where the loop starts) and the **NF** motif in the *Rg*GUS group (i.e., **PAVG**MMRSTR**NF** in *Rg*GUS) and three residues in the *Cp*GUS group (i.e., **PAVG**LHL**NF** in *Cp*GUS) (Fig. 6 and Supplementary Fig. S7). This difference is likely to result in different conformations and possibly different polarities and flexibilities for loop 3. Furthermore, the sequences of loop 5 in the *Rg*GUS, *Bf*GUS, and *Bifd*GUS groups had polar, charged, or non-polar aliphatic residues, whereas non-polar aliphatic and aromatic residues were more common in loop 5 of the *Ec*GUS and *Cp*GUS groups (Fig. 6). Therefore, the gut contains different groups of bacterial GUSs, and enzymes in the same group share a similar ABS sequence and structure. The bacteria in the *Bifd*GUS, *Bf*GUS, and *Rg*GUS groups are part of the normal gut microbiota, whereas those of the *Ec*GUS and *Cp*GUS groups are mostly enteropathogenic and opportunistic pathogens in human and animals.

It appears that at least two broad functional groups of GUSs can be categorized in accordance with our structure-function studies, including the GUSs that are responsible for xenobiotics-induced toxicity and those that are important for maintenance of a healthy interaction between the host and gut microbiota. We have presented several pieces of evidence indicating that GUSs of the first group are expressed by potentially opportunistic and enterobacteria and that the second group of enzymes belongs to commensal gut bacteria. Indeed, small-molecule inhibitors that are more selective toward *Ec*GUS have been found to alleviate irinotecan-induced diarrhea^14^ and intestinal ulcer caused by NSAIDs in mice^15^, suggesting that *Ec*GUS (or a similar GUS) was the main cause. Moreover, treatment with CPT-11 (the prodrug form of SN-38) was found to increase the presence of *Clostridium* cluster XI and *Enterobacteriacea*^42^. In other studies, treatment with CPT-11 increased the abundance of *E. coli, Staphylococcus spp*., and *Clostridium spp*. but decreased the abundance of *Lactobacillus spp., Bifidobacterium spp*., and *Bacteroides* spp. Treatment with this drug also led to an increased level of GUSs in the jejunum and colon^43,44^. Opportunistic or pathogenic bacteria that produce GUSs often exist at a low level in healthy gut microbiota^26^. Therefore, it is important to know the expression levels and activities of GUSs of commensal bacteria. The features of the ABSs in *Bf*GUS, *Rg*GUS, and *Bifd*GUS are conserved in GUSs of a large number of commensal bacteria. As noted earlier, these enzymes prefer a few substrates with one or more polar groups, indicating that their preferred substrates may not be typical xenobiotic glucuronides.

In our NCBI-Blastp search, we noticed that many bacteria found in fermented foods and drinks contain a GUS gene. *Lactobacillus*, *Bifidobacterium*, *Enterococcus*, and *Pediococcus* strains are commonly used or studied as probiotics^45–48^. *Lactobacillus* and *Enterococcus*, isolated from human feces, have been reported to exhibit GUS activity for **17** (phenolphthalein-glucuronide)^49^. A GUS gene exists in the genome of several strains of *L. brevis*, *L. gasseri*, *L. casei*, *L*. *rhamnosus*, and *E. faecalis*. All these GUS sequences contain the longer form of loop 3, except for the GUS, WP_063516954.1, which has an α2-helix loop sequence similar to that of *Bifd*GUS. These observations suggest that the substrate preference of probiotic bacterial GUSs may be strain-specific. The probiotic *E. coli* strain Nissle 1917^50^ expresses a GUS (GenBank: CCQ05100.1) that is 99% identical to the *Ec*GUS that we discussed. Because bacterial GUS activity potentially can affect the pharmacodynamics and pharmacokinetics of certain drugs, patients need to be careful when consuming probiotics and fermented food or drink.

In summary, given that xenobiotic toxicities and drug efficacies might be influenced by the gut microbial GUS composition in the gut and by their substrate specificity, the structure-activity relationship studies of GUSs at molecular level are needed to understand the inter-individual variation of drug responses. By using multidisciplinary approaches, we established the connections between the substrate preferences of GUSs and their ABS structures, flexibilities and hydrophobicities. Notably, we found that the GUSs of enterobacteria and opportunistic bacteria can be key players that cause xenobiotic-induced toxicity in the gut. Conversely, the GUSs of commensal bacteria are less active for toxic xenobiotic glucuronides. We also demonstrated a promising approach to selectively inhibit the actions of undesirable GUSs.

## Methods

### Expression and purification of GUSs

The plasmid pRESTB containing the gene encoding *Ec*GUS was generously provided by the laboratory of Prof. Steve Roffler, IBMS, Academia Sinica. The *Ec*GUS gene was transferred from this plasmid into a pET28b (Novagen) within a frame of N-terminal (His)_6_-tag. The codon-optimized *Rg*GUS, *Bifd*GUS, *Cp*GUS and *Bf*GUS genes that had been individually inserted into plasmid pUC57 were purchased from GenScript. Each gene was then individually transferred into pET15b vector containing (His)_6_-tag nucleotide sequence. GUS mutants were PCR-generated with the wild-type codon for the residue of interest in the GUS cDNA replaced with the mutant codon using a pair of primers and the wild-type gene as the template. The wild-type and mutant proteins were expressed in *E. coli* BL21(DE3) competent cells for 16 h at 16 °C after induction by isopropyl β-D-1-thiogalactopyranoside. The proteins were purified to homogeneity by Ni^2+^ affinity chromatography (HisTrap HP, 5 × 5 ml, GE Healthcare Life Sciences) and gel filtration chromatography (Superdex 200 10/200 GL, GE Healthcare Life Sciences). The purified proteins were concentrated to 50 mg/ml, as determined by Bradford method, and stored at 4 °C in gel filtration buffers: for *Ec*GUS and *Cp*GUS, 20 mM Tris, pH 8.0, 150 mM sodium chloride, for *Bf*GUS, 20 mM Tris, pH 8.0, 150 mM sodium chloride, 10% glycerol and 5 mM β-mercaptoethanol (BME); for *Rg*GUS, 50 mM sodium citrate, pH 6.0, 150 mM sodium chloride, 5 mM BME; and for *Bifd*GUS, 50 mM sodium succinate, pH 6.0, 150 mM sodium chloride.

### Crystallization and structure determination

A Phoenix Liquid Handling System (Art Robbins Instruments) was used to screen the crystallization conditions for *Rg*GUS, *Bifd*GUS, and *Bf*GUS in conjunction with reagents from Structure I and II, Structure Footprint Screen, 3D Structure Screen, Clear strategy, and Macrosol screening kits from Molecular Dimensions, and Wizard I, II, III and IV random sparse matrix crystallization screens and Cryo I and II screen kits from Rigaku in sitting drop plates (Intelli Plates® ARI Molecular Dimensions) at a protein-to-well-solution ratio of 1:1. Diffraction quality crystals of *Rg*GUS (3 mg/ml) and *Bf*GUS (15 mg/ml) were grown in 100 mM sodium citrate buffer, pH 4.3, 100 mM sodium citrate salt, 22% (w/v) PEG 3350 at 4 °C or in 250 mM DL-malic acid, pH 7.0, 20% (w/v) PEG 3350 at room temperature, respectively. The structures of the two proteins complexed with UIFG were obtained by soaking the native crystals with the UIFG for 10 min. To obtain an enzyme-substrate complex structure, we have created the catalytically inactive mutant *Bifd*GUS^E479A^ to avoid substrate hydrolysis. The crystals of *Bifd*GUS^E479A^ (5 mg/ml) were grown in 100 mM sodium cacodylate, pH 6.5, 18% (w/v) PEG 2000 mono-methyl ether at room temperature. Compound **10**, which served as the “substrate”, was soaked into the crystals for 5 min before data collection. The crystals were cryo-protected with the well solution supplemented with 15% (w/v) PEG 200 or 20% (v/v) glycerol and then flash frozen in liquid nitrogen. X-ray diffraction data were collected at National Synchrotron Radiation Research Center, beamline, BL13B1. Diffraction data were indexed and integrated with the HKL2000 program suite^51^. The structures were determined by the molecular replacement using previously resolved structures as the templates. Ligand-free, wild-type *Bifd*GUS and apo *Bf*GUS (PDB code: 3CMG) structures were used as search models for *Bifd*GUS^E479A^/**10** and *Bf*GUS/UIFG complexes, respectively. For *Rg*GUS, the search model was *Ec*GUS (PDB code: **3LPG**). The structures were refined using the Phenix software suit^52^ and COOT^53^. Data collection and refinement statistics of the structures are listed in Supplementary Table S3 (available).

### GUS activity assays

The GUS catalytic activities were measured using absorbance/fluorescence signals of the product aglycones (method 1) or by quantifying the released glucuronic acid with uronic acid dehydrogenase (UDH)-coupled assay (method 2)^54^. Substrates with chromophore and fluorophore aglycones are **4** (410 nm), **5** (460 nm), **17** (540 nm), **13** (400_ext_ nm, 460_em_ nm), **12** (365_ext_ nm, 445_em_ nm), and SN38G (460_ext_ nm, 540_em_ nm) respectively. (Absorbance wavelengths and excitation/emission wavelengths are given in parentheses in the preceding sentence.) In method 1, the assay mixture contained 80 µl of 50 mM sodium citrate, 150 mM NaCl, pH 6.0, 10 μl substrate (in DMSO) and 10 μl enzyme in citrate buffer solution. The reaction was allowed to continue for 10 min at 37 °C, and was then terminated with 100 µl of 500 mM sodium hydroxide, 500 mM glycine, pH 11. Next, the absorbance or fluorescence signal was measured at the corresponding wavelength in a Flex Station 3 Multi-Mode Microplate Reader. For method 2, the amount of released glucuronic acid was quantified by measuring the fluorescence of the NADH generated through action of UDH at 340_ext_ nm (460_em_ nm). The assay mixture was that used for method 1; the GUS reaction proceeded for 10 min at 37 °C, and then 80 μl of 200 mM Tris-HCl, pH 8.0, 10 μl NAD (final concentration, 2 mM) and 10 μl (300 mU) UDH was added. To ensure the oxidation of all glucuronic acid, an excess amount of UDH was used. To calculate the specific activity, the substrate concentration was 250 μM (or 100 μM for drug glucuronides) in the presence of 10 ng or 50 ng of GUSs (*Ec*GUS and *Cp*GUS; 1.42 nM and 1.39 nM, respectively; for *Rg*GUS, *Bf*GUS and *Bifd*GUS, 6.88 nM, 6.59 nM, and 6.48 nM, respectively). To determine the kinetic constants, the enzyme concentration and concentrations of its substrates were first optimized so that the absorbance and fluorescence intensity remained linear. The kinetic constants (K_M_ and V_max_) were calculated by fitting the data to the Michaelis-Menten equation in Prism version 6.0c for Mac (GraphPad Software, La Jolla California USA, www.graphpad.com).

### GUS inhibition assay

The UIFG and UIFG-Pr inhibitory activities for the GUSs were tested in the presence of substrates **4**, **12**, **13**, and **17**. Each inhibitor (1 μM) was pre-incubated for 10 min at 37 °C in 70 μl of 50 mM sodium citrate, 150 mM sodium chloride, pH 6.0, with 10 μl of an enzyme. Then, 10 μl substrate was added into each mixture and the incubation proceeded for an additional 10 min. After the addition of 500 mM sodium hydroxide, 500 mM glycine, pH 11, the fluorescence or absorbance was measured at the wavelength appropriate for the substrate.

### Bioinformatics tools

NCBI Blastp was used to search for GUS sequences. The sequences were aligned with T-Coffee^55^ and the alignment was formatted with BoxShade server (http://www.ch.embnet.org/software/BOX_form.html). The T-coffee-aligned sequences were used to create the sequence logos with WebLogo^56^.

### Docking and MD simulations

The crystal structure of *Cp*GUS (PDB code: **4JKM**) was modified *in silico* to form inactive *Cp*GUS^E412A^. Compound **10** was then docked into the active site of *Cp*GUS^E412A^ (15 × 15 × 15 Å) using the exhaustiveness setting in AutoDock Vina 1.1.2^35^. The initial coordinates of *Cp*GUS^E412A^/**10** complex for MD simulations were obtained from the top-scoring of docking conformations of *Cp*GUS^E412A^/**10**. All MD simulations were performed with GROMACS 2016.3^57^ using the CHARMM22^58^ force field. Apo *Cp*GUS^E412A^ and the docked *Cp*GUS^E412A^/**10** complex were each centered in a 80 × 120 × 150 Å triclinic periodic box filled with TIP3P water molecules. Parameterization of **10** was performed at the SwissParam^59^ server to obtain a format compatible with the CHARMM22 force field. To ensure global charge neutrality of the system, counterions were added to the box as needed. For the interactions that occurred throughout the simulations, an integration step of 2 fs was used. A cutoff of 12 Å was used for van der Waals interactions and Ewald sum electrostatic interactions. A constant temperature of 310 K was maintained using a Berendsen thermostat, and the constant pressure of 1 atm was maintained with a Berendsen Barostat. The systems were equilibrated before the start of the simulations with nvt and npt steps of 100 ps each, with freely moving water molecules, but protein and substrate were constrained. The 20 ns of production phase was then run, and the frames were recorded at 1 ps of intervals. The RMSFs of the backbone atoms were calculated using the gmx rmsf utilities in GROMACS MD package.

### Acquisition and synthesis of xenobiotic glucuronides

Compounds **1**, **4**, **12**, **17**, **18** and morphine-3-glucuronide were purchased from Sigma Aldrich. SN38 glucuronide was purchased from Cayman Chemical. Diclofenac glucuronide was purchased from Toronto Research Chemicals. All other glucuronides were synthesized according to previous reports^28,29^.

## Electronic supplementary material

Supplementary file

## Data Availability

the PDB codes for the coordinates and structure factors of the GUS structures reported in this paper are 5Z18, 5Z19, 5Z1A and 5Z1B. Additional data are provided in the supplemental information file. Spectral data for the synthesized compounds are available upon request.
