# Supplementary material for: β-Glucuronidases of opportunistic bacteria are the major contributors to xenobiotic-induced toxicity in the gut
Source: Sci Rep. 2018 Nov 6;8:16372. doi: 10.1038/s41598-018-34678-z (PMC6219552; doi:10.1038/s41598-018-34678-z)
Supplement: Supplementary file 1 — Supplementary file [file 41598_2018_34678_MOESM1_ESM.pdf]

β-Glucuronidases of opportunistic bacteria are the major contributors to xenobiotic-induced toxicity in the gut

Punsaldulam Dashnyam[1,2,3], Ramesh Mudududdla[2], Tung-Ju Hsieh[2], Ting-Chien Lin[2], Hsien-Ya Lin[2], Peng-Yuan Chen[2], Chia-Yi Hsu[2], and Chun-Hung Lin[1,2,4,5,*]

[1]Molecular and Biological Agricultural Sciences, Taiwan International Graduate Program, Academia Sinica and National Chung-Hsing University, Taipei 11529, Taiwan
[2]Institute of Biological Chemistry, Academia Sinica, Taipei 11529, Taiwan
[3]Graduate Institute of Biotechnology, National Chung-Hsing University, Taichung 40227, Taiwan
[4]Biotechnology Center, National Chung-Hsing University, Taichung 40227, Taiwan
[5] Department of Chemistry and Institute of Biochemical Sciences, National Taiwan University, Taipei 10617, Taiwan
[*]chunhung@gate.sinica.edu.tw

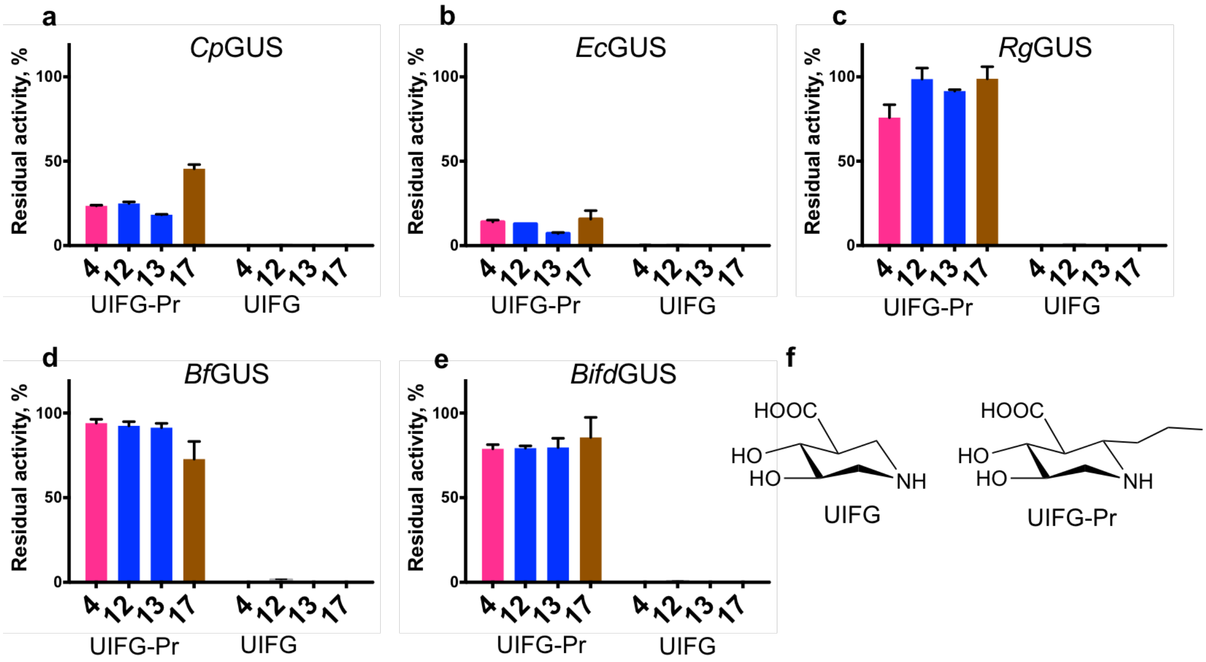

Supplementary figure S1. **UIFG-Pr selectively inhibits *Cp*GUS and *Ec*GUS.** Residual activities of GUSs upon inhibition of 1 uM UIFG and 1 uM UIFG-Pr (panels **a-e**) and molecular structures of the inhibitors (Panel **f**). UIFG universally inhibits the GUSs, whereas UIFG-Pr shows selectivity toward *Cp*GUS and *Ec*GUS.

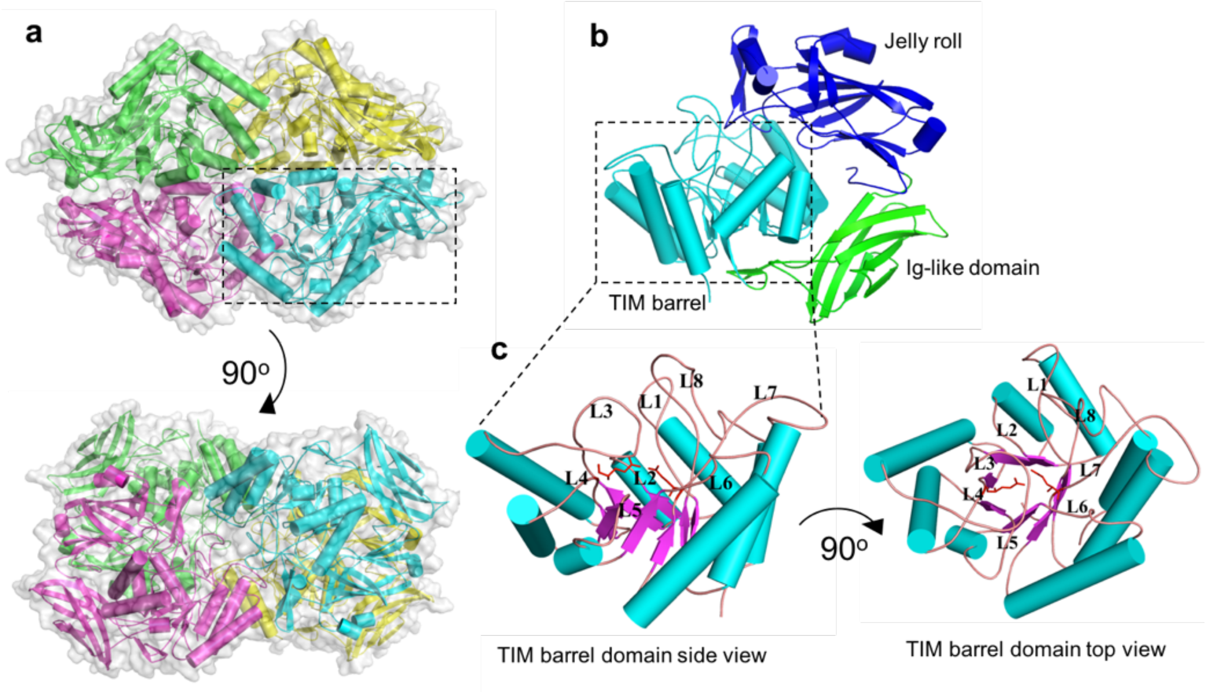

Supplementary figure S2. **Overall tetrameric and tertiary structures of *Rg*GUS.** (**a**) A side and a top view of a *Rg*GUS tetramer. (**b**) Each monomer consists of a N-terminal jelly-roll domain (blue), an immunoglobulin heavy chain-like domain (green), and a C-terminal TIM barrel domain (cyan). (**c**) A side and a top view of the TIM barrel domain that is composed of eight α-helices (cyan) and eight β-sheets (magenta). These secondary structures are connected by α/β and β/α loops (wheat) that constitute substrate-binding site of the GUS. These loops are numbered from 1 to 8 starting from the N-terminus of the TIM barrel domain.

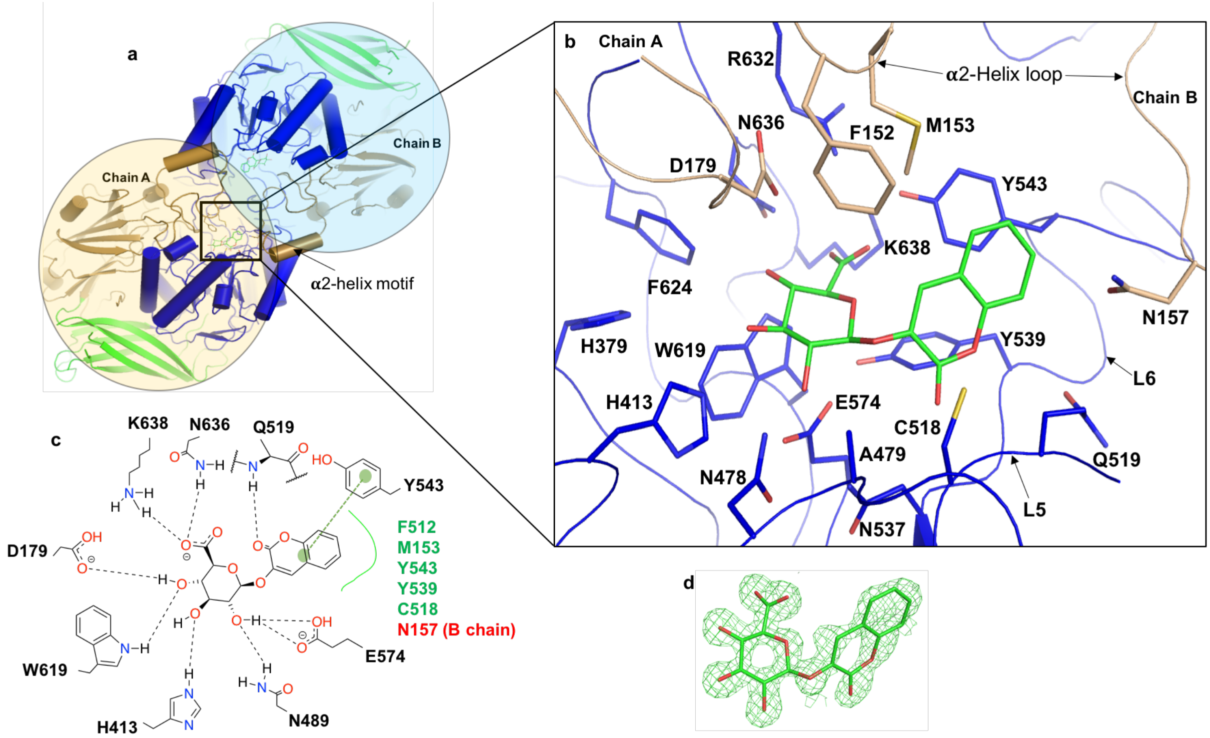

Supplementary figure S3. A substrate-binding site of *Bifd*GUS. (**a**) A dimer of tetrameric *Bifd*GUS^{E479A} complexed with substrate **10**. A tertiary structure of the enzyme consists of a jelly-roll (wheat), an immunoglobulin heavy chain-like (green), and a TIM barrel domain (blue). (**b**) A close-up image of the *Bifd*GUS^{E479A}/**10**. **10** is shown as green stick. The amino acid residues (shown as sticks) interacting with **10** are shown in blue and wheat sticks Aglycone-binding site of the enzyme is formed by α2-helix loops of the two adjacent chains (shown as A and B chains), loop 5, and loop 6. Loops 5 (L5) and 6 (L6) are at the TIM barrel domain; the α2-helix loop is resided at the jelly-roll domain. Panel **b** shows the aglycone-binding site of A chain. Residues F152 and M153 are from the α2-helix loop at the jelly-roll domain of A chain, residue N157 is from the α2-helix loop at the jelly-roll domain of B chain. Residue D179 that makes a hydrogen bonding with 4-OH of glucuronic acid moiety of **10** locates at the jelly-roll domain. (**c**) 2D representation shows the important interactions between *Bifd*GUS^{E479A} and **10**. (**d**) 2Fo-Fc omit map of **10** countered at σ level=1, carved at 1.6.

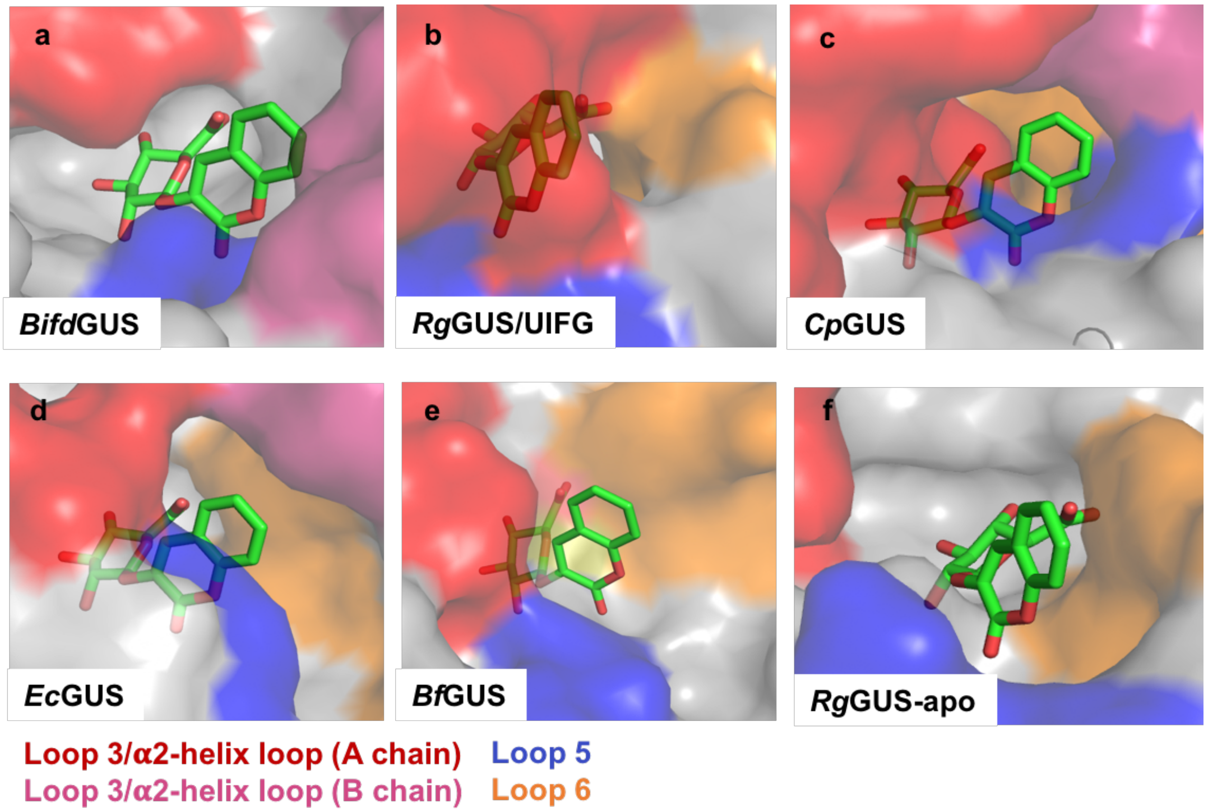

Supplementary figure S4. **Surface presentations showing the active site architectures of the five bacterial GUSs.** *Cp*GUS (**c**) and *Ec*GUS (**d**) have similar small aglycone-binding sites. The aglycone-binding sites of *Bf*GUS (**e**) and *Bifd*GUS (**a**) are relatively larger because their loops 3 are shorter than that of the former two enzymes. Tip of loop 3 is ordered in *Rg*GUS/UIFG complex (**b**), but it is disordered in apo *Rg*GUS as shown in panel **f**. **10** is shown as green sticks. Images in panels **b-f** were produced by superimposing them on the *Bifd*GUS^{E479A}/**10** complex, which is not shown for clarity reason.

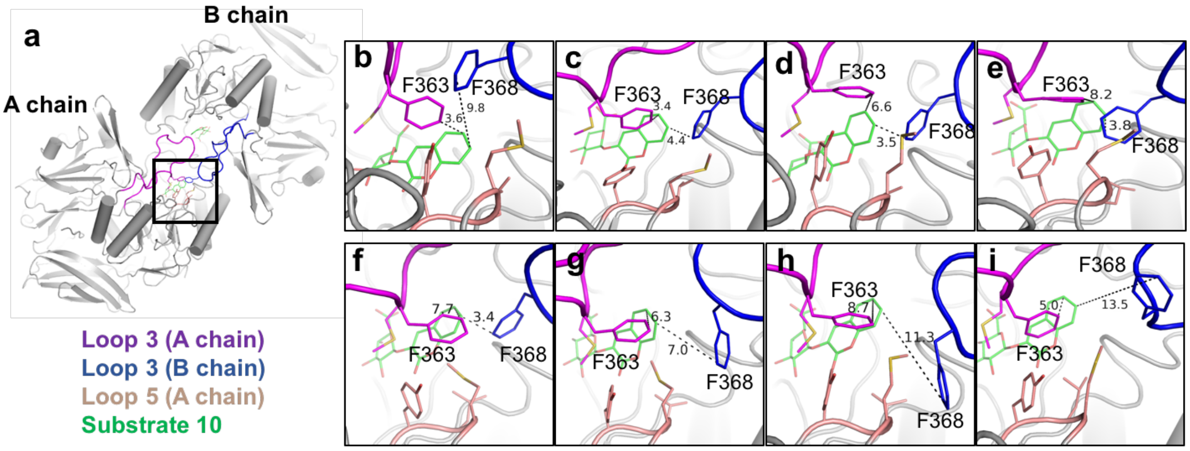

Supplementary figure S5. Flexibility at the aglycone-binding site of *Cp*GUS. (**a**) A dimeric structure of *Cp*GUS. Loops 3 of A and B chains are colored in magenta and blue, respectively (**b**)-(**i**). Distance between F363 (A chain) and F368 (B chain) to C6 and C7 of **10**, respectively, fluctuated during the MD simulations.

a

α2-helix loop

```
WP_003467686.1 (CpGUS)    134 NIIDETTLPVGL-------------VKEVEVD---GKKVIK--NSVNFDF
WP_085460699.1 (EcGUS)    136 NELNWQTIPPGM-------------VITDE------NGKKK--QSYFHDF
AAQ76046.1 (RgGUS)        140 NELNETSLPCGA-------------TKILN----NGRKLAK--PY--FDF
WP_012902563.1 (BifdGUS)  135 NRIGSSTLPVGNDAGTAFMGSDNANVPAV-AE---AKKHARRQNLPNFDF
CAH06099.1 (BfGUS)        120 NGEQLDIMPL--------------------V----------------GDF
NP_000172.2 (HsGUS)       174 NTLTPTTLPPGT-------------IQYLTDTSKYPKGYFV--QNTYFDF
```

b

Loop 1

```
WP_003467686.1 258 LD---D---E-E---IIDTYFEEFGVRTVEVKDG-KFLINNKPFYFKGFGKHEDSYVNGR
WP_085460699.1 257 KS---Q-----T---ECDIYPLRVGIRSVAVKGE-QFLINHKPFYFTGFGRHEDADLRGK
AAQ76046.1     261 TD-------GNG---VLDEYREKIGIRTVRI-EGTKILLNDRPVYLKGFGKHEDFPILGR
WP_012902563.1 333 LP---EGGAE-SSSRIIDAYRQTFGIRTVEVSGT-TFLINGKPFYFKGFGKHEDSYFHGR
CAH06099.1     250 HK---D----GK---QIDSVTQPLGLRYYHTDPDKGFFLNGKHLPLHGVCRHQDRAEVGN
NP_000172.2    308 TAQTSL---G-P---VSDFYTLPVGIRTVAVTKS-QFLINGKPFYFHGVNKHEDADIRGK
```

Loop 2

```
WP_003467686.1 307 GINEAINIK-DFNLMKWIGANSFRTSHYPYSEEIMRLADREGIVVIDETPAVGLH----L
WP_085460699.1 305 GFDNVLMVH-DHALMDWIGANSYRTSHYPYAEEMLDWADEHGIVVIDETAAVGFN----L
AAQ76046.1     310 GFHWGI-VKRDFECLKWTNANCFRTSHYPYAEEWYQFADEEGFLIIDEVPAVGMMRSTR-
WP_012902563.1 388 GTDDVLNVK-DVSLIHWLHANSFRTSHYPYAESMYDLCDREGIVIIDEVPAVGMS---WL
CAH06099.1     300 ALRPQHHEE-DVALMREMGVNAIRLAHYPQATYMYDLMDKHGIVTWAEIPFVGPG-----
NP_000172.2    360 GFDWPLLVK-DFNLLRWLGANAFRTSHYPYAEEVMQMCDRYGIVVIDECPGVGLA-----
```

Loop 3

```
WP_003467686.1 362 NFMATG--FGG-----DAPKRDTWKE-IGTKEAHERILRELVSRDKNHPCVVMWSVANEP
WP_085460699.1 360 SLGIGFEAGNK-----PKELYSEEAVNGETQQAHLQAIKELIARDKNHPSVVMWSIANEP
AAQ76046.1     368 NFVAAG--SGNYTYFFEAL--T-VPEL--LK-SHIADTEEMITRDKNHPSVIAWSLFNEP
WP_012902563.1 444 QY-AN-------------PL-------V--AERHREAIRGMIARDKNHPCIVMWSIANEP
CAH06099.1     354 -----------------GYADKGFVDQ-ASFRENGKQQLIELIRQHYNHPSICFWGLFNEL
NP_000172.2    414 ---------------------LPQFFN-NVSLHHHMQVMEEVVRRDKNHPAVVMWSVANEP
```

Loop 4 Loop 5

```
WP_003467686.1 414 DSDSEG-----AKEYFEPLIKLTKELDPQKRPVTVVTYLM----STPDRCKVGD-IVDVL
WP_085460699.1 415 DTRPQG-----AREYFAPLAEATRKLDPT-RPITCVNVMF----CDAHTDTISD-LFDVL
AAQ76046.1     420 ETITDY-----AYEYFKEVFAAAETYDFQSRPMTGAFEKN----SKPELCKCYP-LCDFI
WP_012902563.1 481 GLDGDGERPRQAYDYFRPLYELAHASDPQNRPVTLVCCQNDYT---TD---ITERTMDVV
CAH06099.1     397 KEVGDN-----PVEYVKELNALAKQEDPT-RPTTSASNQDG---------NLNF-ITENI
NP_000172.2    453 ASHLES-----AGYYLKMVIAHTKSLDPS-RPVTFVSN------SNYAADKGAP-YVDVI
```

Loop 6 Loop 7

```
WP_003467686.1 464 CLNRYYGWYVAGG-DLEEAKRMLEDELKGWEERCPKTPIMFTEYGADTVAGLHDTVP-VM
WP_085460699.1 464 CLNRYYGWYVQSG-DLETAEKVLEKELLAWQEKLH-QPIIITEYGVDTLAGLHSMYT-DM
AAQ76046.1     470 CLNRYYGWYISGGPEIEEAEELFRDEMDRWKAKELNVPFVFTEFGTDTMAGLHK-LPSIM
WP_012902563.1 535 CINRYYGWYNLSG-DLDAACHALNIELDFWEN-IGK-PVMFTEYGADTIEGIHGTHG-EM
CAH06099.1     441 AWNRYDGWYGS-------TPKTLATFLDRTHKKHPELRIGISEYGAGASIYHQQDSL-KQ
NP_000172.2    500 CLNSYYSWYHDYG-HLELIQLQLATQFENWYKKYQ-KPIIQSEYGAETIAGFHQDPP-LM
```

Loop 8

```
WP_003467686.1 522 -------FTEEYQVEYY-KANHEVMDKCK--NFV-GEQVWNFADFATSQGII--RVQGNK
WP_085460699.1 521 -------WSEEYQCAWL-DMYHRVFDRVS--AVV-GEQVWNFADFATSQGIL--RVGGNK
AAQ76046.1     529 -------WSEEYQKEYL-EMNFRVFDSYE---FVQGELAWNFADFQTTEGIM--RVDGNH
WP_012902563.1 591 -------FSEEFQRDYYARINAEI-DKRP--WFI-GEQLWNFADFATFQGII--RVEGNR
CAH06099.1     493 PSASGWWHPENWQTYYH-MENWKIIAERP--FVW-GTFVWNMFDFGAAHRTEGDRPGIND
NP_000172.2    557 -------FTEEYQKSLL-EQYHLGLDQKRRKYVV-GELIWNFADFMTEQSPT--RVLGNK
```

```
WP_003467686.1 569 KGIFTRER-KPKMIAHSLRERWTNIPEFGYKK---------------------------
WP_085460699.1 568 KGIFTRDR-KPKSAAFLLQKRWTGMNFGEKPQQGGKQ-----------------------
AAQ76046.1     576 KGVFTRDR-QPKAAAVVFKDRWEKKNELF-------------------------------
WP_012902563.1 638 KGILTRDR-QPKMAAHWLRERWAGIPDYGYKG---------------------------
CAH06099.1     549 KGLVTFDRKVRKDAFYFYKANWNKQEPMIYLAEKRCRLRYQPEQTFMAFTTAPEAELFVN
NP_000172.2    606 KGIFTRQR-QPKSAAFLLRERYWKIANETRYPHSVAKSQCLENSLFT-------------
```

Supplementary figure S6. Sequence alignment of five bacterial GUSs and human GUS showing (**a**) α2-helix loop region of N-terminal domain and (**b**) C-terminal domain.

(a) The *Bifd*GUS group: α2 helix loop

```
WP_012902563.1  130  DNRIGSSTLPVGNDA-GTAFMGSDNANVPAVA---EAKKH---------------AR-RQN
WP_034874654.1       DNRIGSSTLPVGNDA-GTAFMGSDNANVPAVA---EAKKH---------------AR-RQN
CBL12719.1           DNRINHSTLPVGNEE-GTSFMGADNAGVPGVEAAKRWNEEGTSFMGADNAGVPGVERKPQN
WP_007715113.1       DNRISHSTLPVGNEG-TTAFFGSDNPGVPSV----EAAKKW-------------R--GQQN
SEU10886.1           DNRISHSTLPVGNEG-TTAFFGSDNPGVPSV----EAAKKW--------------R-GQQN
WP_024735652.1       DNRISHSTLPVGNEG-TTAFFGSDNPGVPSV----EAAKKW------------R---GQQN
WP_006863968.1       DNHVNHSTLPVGNES-ATAFFGSDNPGVPSV----EAAKLWR---------------RPQN
CUO31707.1           DNRVNHSTLPVGNEG-STAFFGSDNPGVPSV----EAAKLWR---------------KPQN
WP_005943354.1       DNRVNHSTLPVGNEDGQLAFFGSDNAGIPSV----EAAKRA-----------AA----PQN
WP_065547790.1       DNRLNHSTLPAGNDPKETAFFGSDNAGIPSV----EQAKRE---------QKLQ------N
WP_002587560.1       DNRLNHSTLPAGNDPTETAFFGSDNAGIPSV----EQAKRE---------QKLQ------N
WP_044994201.1       NNRVDHHTLPMGNEEG-TAF-GSDNAQVPSV----QKAKEHRGIV---------------N
SFG50938.1           NNRVDHHTLPMGNEDG-TAFFGSDNAQVPSV----QMAKEHRGIV---------------N
WP_087384128.1       DNRVNRNTLPVGNEPGEIAFFGSDNAGIPSV----EHAKAN-AK--------------PMN
```

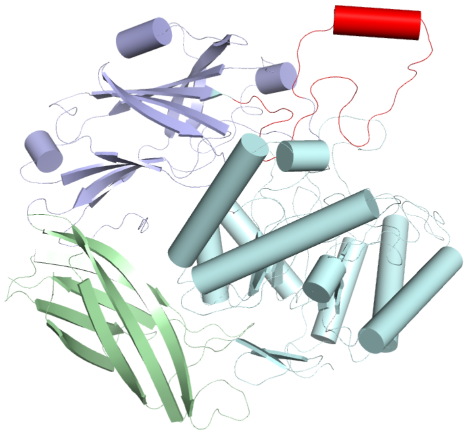

(b) The *Ec*GUS group: Loop 3

```
WP_085460699.1  315  IDETAAVGFNLSLGI-GFEAG-NKPKELYSEEAVNGETQQAH
OUZ60539.1           IDETAAVGFNLSLGI-GFEAG-NKPKELYSEEAVNGETQQAH
WP_039064923.1       IDETAAVGFNLSLGI-GFEAG-NKPKELYSEEAVNGETQQAH
WP_080224385.1       IDETAAVGFNLSLGI-GFDSC-EKPKALYSEEGVNDETQQAH
WP_039046752.1       IDETAAVGFNISLGI-GFDSC-EKPKALYSEEGVNDETQRAH
WP_032697836.1       IDETAAVGFNLSLGI-GFDAC-EKPTSLYGDDAIDDRTQQAH
WP_034458256.1       INETAAVGFNLSLGIT-FDAG-ERPKELYSEEAINGETQQAH
WP_049560671.1       INETPAVGFNLALGLN-FDSV-QKPKELFSDEAVNGQTQQAH
WP_077173911.1       INETPAVGLNLALGFN-FDVT-QKPANLFSEEAINGQTQQAH
```

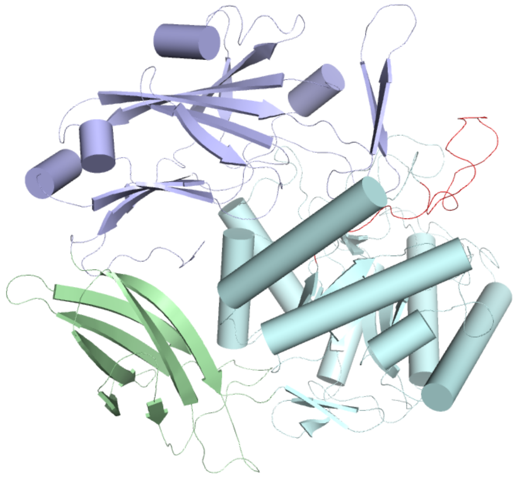

(c) The *Cp*GUS group: Loop 3

```
WP_003467686.1  301  IDETPAVGLHLNFMATG--FGGDAPKRDTWKEIGTKEAHERIL
WP_008680773.1       IDETPAVGVHLNFMATG--FGGDAAKRDTWKEIRTKEAHEQVL
WP_005212204.1       IDETPAVGLHLNFMATGL-FGDSV-KRDTWKEIGTKEAHEQVL
WP_045726255.1       IDETPAVGLHLNFMATGGLFGGGV-KRDTWKEIGTKEAHEQIL
WP_066874435.1       IDETPAVGLHLNFMATGGLFGGGV-KRDTWREIGTKQAHEQVL
WP_002598286.1       IDETPAVGLHLNFMATG-LFGSGV-KRDTWKEIQTKEAHEQVI
WP_074079011.1       IDETPAVGVHLNFMAT--MMAGAE-KRDTWKEIQTFEHHKDII
WP_039680969.1       IDETPAVGVHLNFMAT--MMAGAE-KRDTWKEIQTFEHHKDII
WP_055069253.1       IDETPGVGLHLNFMAT--LLSGDSKKRETWKEIGTKEHHEQVL
WP_073119987.1       IDEVPAVGLHLNFMAT--LLDDNVEKHNTWKEIRTENSHKTVI
WP_060814749.1       IDEVPAVGLHLNFMAT--LLDDNVEKHNTWKEIRTEDSHKTVI
WP_054027879.1       IDETPAVGLHLNFMAA--MFGGGA-KRNTWAELKTQEHHQEII
AAK29422.1           IDETPAVGVHLNFMATT-GLGEGSERVSTWEKIRTFEHHQDVL
WP_068783612.1       IDEVAAVGLHLNFMA----IHSNGPKRNTWKEIRTHEAHRQAI
ARB39939.1           IDETTAVGVHLNFSAA---LDGSQ-NTDTWKKIRTKEDHEQVI
WP_037589849.1       IDETTAVGVHLNFSAA---LDGSQ-NTDTWKKIRTKEDHEQVI
WP_037558698.1       IDETTAVGVHLNFMVA---LGGQL-EHDTWKEIDTHQAHKEVI
```

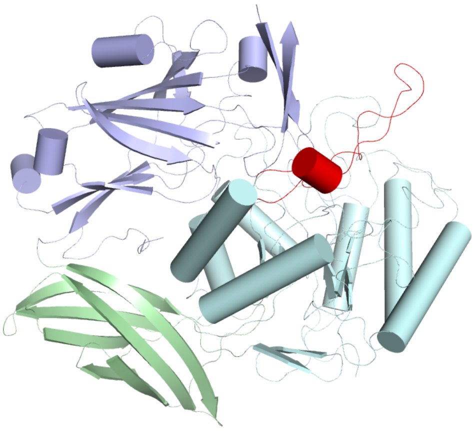

(d) The *Rg*GUS group: Loop 3

```
AAQ76046.1     357  IDEVPAVGMMRSTRNFVAA--GSGNYTYFFEA--LTV--PEL--LKSH
WP_026889983.1      IDEVPAVGMMRSTRNFAAA--GTGQYTGFFET--PTV--PNL--QKAH
WP_024835204.1      IDEVPAVGMMRSTRNFAAA--GTGKYTGFFET--PTV--PNL--QKAH
WP_029471552.1      IDEVPAVGLFESLMNFMEASTGK-KT-AFFAK--DTI--PEL--LENH
WP_018595336.1      IDEVPAVGLFESLMNFMEASTGK-KT-AFFAK--DTI--PELL--ENH
WP_087417720.1      IDEVPAVGLFESLMNFMEAST---KATAFFEKET----TPVL--LQAH
P_027217722.1       IDEVPAVGMMRSTHNFVDAGTG--KYTYFFETP-TV---GTL--QKNH
WP_034927075.1      IDEVPAVGMMRSLMNFAAAGAG-GKDTRFFT-ADTV---PQL--QANH
WP_087020307.1      IDEVPAVGMFPSLMNFTDASSG--KQTAFFAQ-ETA---PEL--QKNH
WP_026510287.1      IDEVPAVGMMRSFFNFMDAGSGG-KYTYFFE---T-DTVPQL--QKNH
WP_077611355.1      IDEVAAVGLFKSTKNFVDASNG--KMSAFFEADTI----PRL--LTRH
WP_027295337.1      IDEVAAVGFFESLMNFMEASTG--KQTAFFSREIVQTE-T-K---ANH
WP_070041796.1      IDEVAAVGFFESLMNFMEASTG--KQTAFFSREIVQTE-T-K---ANH
CVH74462.1          IDELPAVGFFESLMNFLDASGG--KSTQFFER-ETI--PEL-K--KNH
WP_081832803.1      IDEVPAVGMMRSTHNFVDAGTG--KYTYFFETP--TV--PKL--KKNH
```

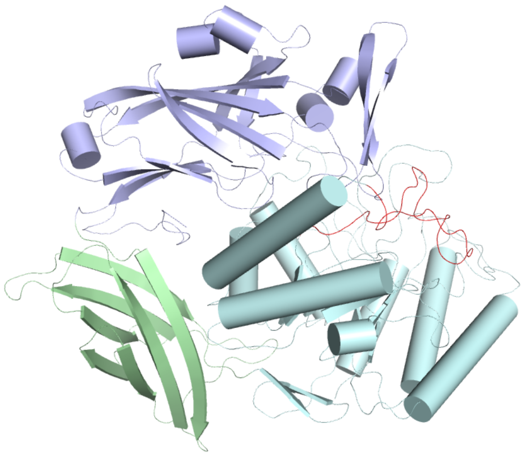

(e) The *Bf*GUS group: Loop 3

```
WP_010991991.1  335 KHGIVTWAEIPFVGPGGYADKGFVDQASFRENGKQQLIELIRQHYN
WP_042369167.1      KYGIITWAEIPFVGPGGYADKGFVDQTSFRENGKLQLIELIRQHYN
WP_073345524.1      KNGIIVWAEIPFVGPGGYNDKGFVDLPAFRANGKEQLKELIRQHYN
WP_061448150.1      KNGIIVWAEIPFVGPGGYNDKGFVDLPAFRANGKEQLKELIRQHYN
WP_008765620.1      KNGIIVWAEIPFIGPGGYDDKGFVNLPSFRANGKEQLKELIRQHFN
WP_005851651.1      KHGIVTWAEIPFVGPGGYADKGFVDQPSFRENGKEQLKEMIRQHYN
WP_005937452.1      RNGIVTWAEIPFVGPGGYADKGFVDQPSFRENGKEQLKEMIRQHFN
CDF16335.1          RNGIVTWAEIPFVGPGGYADKGFVDQPSFRENGKEQLKEMIRQHFN
WP_016277820.1      KNGIVTWAEIPFVGPGGYADKGFVDQPSFRENGKEQLKEMIRQHYN
WP_008861178.1      QSGIITWAEIPFIGPGGYADKGFIDSEAFKKNGEEQLKELIRQNYN
WP_007840419.1      KYGIVTWAEIPFVGPGGYADKGFVDQPSFRENGKEQLKEMIRQHYN
OKZ42125.1          QSGIITWAEIPFVGPGGYADKGFIDSEAFKKNGEEQLKELIRQNYN
CCX61975.1          RYGIIVWAEIPFVGPGGYADKGFVDLPDFRANGKEQLKELIRQHGN
WP_097531265.1      RYGIIVWTEIPFVGPGGYMDKGFVNLPTFRSNGKEQLRELIRQHCN
WP_022021795.1      RYGIIVWAEIPFVGPGGYADKGFVDLPDFRANGKEQLKELIRQHGN
WP_013545761.1      RHGIVTWAEIPFVGPGGYADKGFVDQSSFKENGREQLREMIRQHFN
RGM39504.1          KTGIIAWAEIPFVGPGGYDDKGFVDFPDFRENGKNQLRELIRQNYN
WP_075556066.1      KYGIVTWAEIPFIGPGGYLDRGYNDLPAFRANGKEQLKELIRQHYN
CCY15266.1          RYGFIVWAEIPFVGPGGYSDEGFVNLPAFRQNGRQQLVELIRQHYN
WP_048315356.1      KYGIVAWAEIPFIGPGGYQDRGFNDLPSFRENGKEQLKELIRQHYN
WP_076931253.1      KNGLIVWAEIPFIGPGGYAEQGYVNQPSFRENGKEQLIELIRQHYN
WP_036874743.1      KHGMVVWAEIPFVGPGGYADKGFVDLPTLKANARQQLKELIRQNYN
WP_008154342.1      KYGIVTWAEIPQIGPGGYQDRGYINQPSFRENGKEQLKELIRQHYN
WP_005647062.1      KYGIVTWAEIPQIGPGGYQDQGYINQPSFRENGKEQLKELIRQHYN
WP_055097554.1      KYGLVVWAEIPFVGPGGYQDQGFVNTSFFRQNGKEQLIELIRQHYN
WP_044224780.1      KYGLVVWAEIPFVGPGGYQDQGFVNTSFFRQNGKEQLIELIRQHYN
CCY35738.1          KYGLVVWAEIPFVGPGGYQDQGFVNTPSFCQNGKEQLIELIRQHYN
WP_051305258.1      RTGIVTWAEIPFVGPGGYADKGFVDTERFRDNGKQQLRELIRQNYN
WP_091905861.1      KNGLIVWAEIPFIGPGGYAEQGYVNQPSFRKNGKQQLTELIRQHYN
OJV91079.1          KGGIVVWAEIPFIGPGGYQDRGFINLPSFKANGIEQLRELIRQHYN
WP_004314238.1      KLGIVVWAEIPFIGPGGYEDKGFVDSPSFRANGKEQLKELIRQHYN
WP_008565720.1      RAGIVTWAEIPFVGPGGYADKGFVDSEAFRANGKQQLRELIRQHFN
WP_007135257.1      RAGIVTWAEIPFVGPGGYADKGFVDSKAFRDNGKQQLIELIRQQFN
WP_050756901.1      RAGIVTWAEIPFVGPGGYADKGFVDSERFRANGKAQLCELIRQHFN
WP_066630907.1      KAGFVTWSEIPFVGPGGYRDKGFVNQESFKDNGKQQLIEMIRQQYN
WP_072876176.1      KAGLVVWSEIPFVGPGGYRDKGFVNQASFKENGRTQLKEMIYQQYN
WP_009137415.1      EYGFIVWAEIPFVGPGGYRDKGFVDQLSFRENGKIQLKELIRQNYN
```

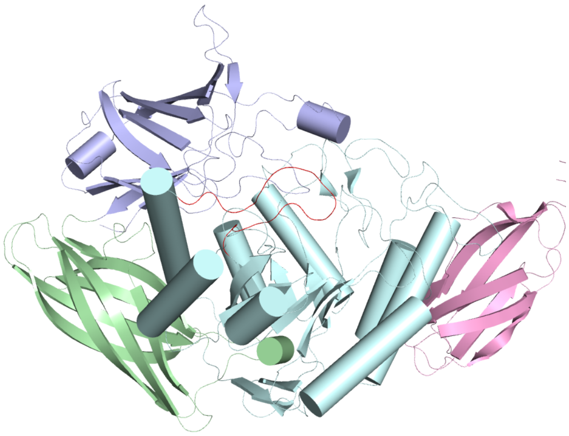

Supplementary figure S7. Sequence alignment of bacterial GUSs. (a) Sequences of α2-helix loop in the *Bifd*GUS group, (**b**) Sequences of loops 3 of the *Ec*GUS, (**c**) the *Cp*GUS (**d**) the *Rg*GUS, and (**e**) the *Bf*GUS groups. Tertiary structures of *Bifd*GUS, *Ec*GUS, *Cp*GUS, *Rg*GUS, and *Bf*GUS are followed each sequence alignment. Light blue, light green, light cyan, and light pink colors represent a N-terminal jelly-roll domain, a middle region immunoglobulin heavy chain-like domain, a TIM barrel domain, and a C-terminal carbohydrate binding domain, respectively. The sequences shown in the sequence alignments are highlighted in red color in the respective 3D structures.

Supplementary Table S1. The kinetic parameters of bacterial GUSs.

		*Bifd*GUS	*Rg*GUS	*Cp*GUS	*Ec*GUS	*Bf*GUS	[1]*Cp*GUS^{RgGUSL5}	*Cp*GUS^{M448F}
**4**	K_M (uM)	1359±227.7	9320±2700	160.6±0.4	201.0±12.9	2402±604	289.7±15.9	242.3±11.8
	k_{cat} (s^{-1})	112.6±8.2	24.7±5.8	139.0±3.9	212.8±4.5	35.3±6.0	23.9±0.2	162.5±0.8
**5**	K_M (uM)	715.6±0.8	1096±273	134.9±15.4	190.4±10.5	398.8±38.3	ND	ND
	k_{cat} (s^{-1})	3.1±0.4	13.6±2.1	33.1±2.5	76.8±1.5	40.4±1.7	ND	ND
**10**	K_M (uM)	1175.0±45.3	930.6±270.6	33.5±0.5	74.8±3.8	N/a	ND	ND
	k_{cat} (s^{-1})	19.4±0.6	34.7±7.4	112.6±0.9	41.1±1.4	N/a	ND	ND
**12**	K_M (uM)	646.0±55.9	2431.8±612.0	89.4±0.6	116.7±1.7	98.8±1.8	57.7±0.8	233.1±21.1
	k_{cat} (s^{-1})	17.2±1.5	11.2±2.2	59.0±1.5	79.9±0.4	9.3±0.1	8.4±0.8	86.4±21.1
**17**	K_M (uM)	363.5±5.9	51.4±22.9	40.6±2.2	23.0±12.9[a]	ND	245.0±44.5	44.5±0.1
	k_{cat} (s^{-1})	55.4±2.0	0.6±0.1	54.1±1.6	0.5±0.1[a]	ND	1.6±0.1	4.6±0.0
**18**	K_M (uM)	ND	117.6±4.4	54.7±2.5[a]	ND	ND	146.6±13.2	N/a
	k_{cat} (s^{-1})	ND	0.7±0.0	0.2±0.0[a]	ND	ND	1.1±0.1	N/a

N/a-Not available
ND-Not detectable (activity is too low for kinetics experiment)
[a]Estimated by using high concentration of enzymes
[1]Original sequence 446 **YLM** of loop 5 of *Cp*GUS is replaced by the sequence 452 **EKN** of loop 5 of *Rg*GUS.

Table S2. The catalytic efficiency (k_{cat}/K_M; 1/s•mM) of bacterial GUSs.

Substrates	**4**	**5**	**10**	**12**	**17**	**18**
*Bifd*GUS	82.9	4.4	16.5	26.6	152.4	ND
RgGUS	2.7[a]	12.4	37.8	4.6	12.9	5.65
*Cp*GUS	865.5	363.7	956.9	659.9	1332.5	3.1
*Ec*GUS	1058.7	403.4	549.7	684.7	21.7[a]	ND
*Bf*GUS	14.7	101.3	N/a	94.1	ND	ND
*Cp*GUS$^{Rg\mathrm{GUSL5}}$	82.7	N/a	N/a	145.6	6.5	7.5
*Rg*GUS$^{Cp\mathrm{GUSL5}}$	57.7	N/a	N/a	5.98	N/a	ND
*Cp*GUS$^{\mathrm{M448F}}$	671.5	N/a	N/a	370.8	103.4	N/a

ND-Not detectable (activity is too low for kinetic experiment)
N/a-Not available
[a]Estimated by using high concentration of enzymes

Supplementary Table S3. X-ray data collection and refinement statistics

	***Bf*GUS/UIFG (5Z1A)**	***Rg*GUS apo (5Z18)**	***Rg*GUS/UIFG (5Z19)**	***Bifd*GUS^{E479A}/Coum-3-G (5Z1B)**
Data collection				
Space group	I222	C121	C121	P1211
Cell dimension				
a, b, c (Å)	80.253, 103.135, 199.147	181.868, 118.182, 210.355	181.282 112.359 209.795	91.34 104.165 160.673
α, β, γ (deg)	90, 90, 90	90, 93.45, 90	90 93.45 90	90 90.4 90
Resolution (Å)	29.40 -1.86 (1.93-1.86)	29.03-2.495 (2.585-2.495)	29.92-2.503 (2.593-2.503)	29.16-1.45 (1.502-1.45)
I/s	26.17 (3.57)	11.14 (3.86)	6.49 (1.74)	27.36 (1.90)
Completeness (%)	99.1 (98.3)	98.9 (99.2)	98.9 (97.3)	99.5 (97.8)
Redundancy	4.2 (3.6)	3.8 (3.9)	3.2 (2.6)	3.6 (3.2)
Refinement				
R_{work}/R_{free}	0.1578/0.1895	0.1672/0.2094	0.1842/0.2187	0.1301/0.1602
B factors (mean)	22.74	36.50	42.12	21.60
Macromolecules	21.08	36.40	42.15	20.40
Ligand	16.02	-	38.23	26.90
Water	33.34	38.20	41.20	31.70
R.m.s deviations				
Bond lengths (Å)	0.005	0.009	0.004	0.012
Bond angles (deg)	0.98	1.24	0.99	1.40
Ramachandran favored (%)	97	97	96	97
Ramachandran outliers (%)	0.00	0.086	0.31	0.041
